# Functional MOF-Based Materials for Environmental and Biomedical Applications: A Critical Review

**DOI:** 10.3390/nano13152224

**Published:** 2023-07-31

**Authors:** Maria-Anna Gatou, Ioanna-Aglaia Vagena, Nefeli Lagopati, Natassa Pippa, Maria Gazouli, Evangelia A. Pavlatou

**Affiliations:** 1 Laboratory of General Chemistry, School of Chemical Engineering, National Technical University of Athens, Zografou Campus, 15772 Athens, Greece; 2 Laboratory of Biology, Department of Basic Medical Sciences, Medical School, National and Kapodistrian University of Athens, 11527 Athens, Greece; iliannavgn@med.uoa.gr (I.-A.V.); nlagopati@med.uoa.gr (N.L.); mgazouli@med.uoa.gr (M.G.); 3 Biomedical Research Foundation, Academy of Athens, 11527 Athens, Greece; 4 Section of Pharmaceutical Technology, Department of Pharmacy, School of Health Sciences, National and Kapodistrian University of Athens, 15771 Athens, Greece; natpippa@pharm.uoa.gr; 5 School of Science and Technology, Hellenic Open University, 26335 Patra, Greece

**Keywords:** MOFs, porous materials, environment, heavy metals, biomedicine, cancer

## Abstract

Over the last ten years, there has been a growing interest in metal–organic frameworks (MOFs), which are a unique category of porous materials that combine organic and inorganic components. MOFs have garnered significant attention due to their highly favorable characteristics, such as environmentally friendly nature, enhanced surface area and pore volume, hierarchical arrangements, and adjustable properties, as well as their versatile applications in fields such as chemical engineering, materials science, and the environmental and biomedical sectors. This article centers on examining the advancements in using MOFs for environmental remediation purposes. Additionally, it discusses the latest developments in employing MOFs as potential tools for disease diagnosis and drug delivery across various ailments, including cancer, diabetes, neurological disorders, and ocular diseases. Firstly, a concise overview of MOF evolution and the synthetic techniques employed for creating MOFs are provided, presenting their advantages and limitations. Subsequently, the challenges, potential avenues, and perspectives for future advancements in the utilization of MOFs in the respective application domains are addressed. Lastly, a comprehensive comparison of the materials presently employed in these applications is conducted.

## 1. Introduction

Porous materials play a crucial role in various applications such as gas storage, separation of gases/vapors through adsorption, selective catalysis based on shape and size, the storage and release of drugs, as well as serving as templates for synthesizing low-dimensional materials [1,2,3,4,5,6]. Conventionally, porous materials are categorized as either organic or inorganic. Among the organic options, activated carbon is the most commonly utilized porous material. These materials are typically derived from carbon-rich sources through a process called pyrolysis. They exhibit remarkable characteristics such as large surface areas and enhanced adsorption efficiencies, albeit lacking well-defined structures. In spite of their lack of order, porous carbon materials find widespread applications, entailing gas separation and storage, water purification, and solvent extraction and recovery [7].

Inorganic porous frameworks exhibit well-organized arrangements, exemplified by zeolites. Their synthesis typically involves the use of an inorganic or organic template, which establishes strong interactions with the inorganic framework during the synthesis process. Consequently, the removal of the template can lead to the collapse of the framework. In terms of element composition, inorganic frameworks often lack diversity, with the elements employed primarily revolving around Al, Si, and chalcogens. Despite these limitations, inorganic frameworks have proven valuable in applications related to separation and catalysis [3].

Over the past few decades, significant attention has been devoted to the evolution and thorough investigation of metal–organic frameworks (MOFs) that constitute porous hybrid materials designed to combine the advantageous characteristics of both organic and inorganic porous materials. These materials consist of organic ligands serving as struts and coordinated metal ions/ion clusters acting as nodes. By manipulating the vast array of possible combinations between metal nodes and organic ligands, MOFs with diverse characteristics can be readily synthesized. These characteristics include an extensive variety of pore sizes (from micropores to mesopores or macropores), rigid or flexible skeletons, and adjustable surface affinities, all of which greatly influence the properties of MOFs [2]. Furthermore, factors such as particle morphology, size distribution and chemical properties are crucial in shaping the applications of MOFs. Leveraging their advantageous features, such as facile synthesis, customizable pore sizes and structure, numerous active sites, diverse functional groups, variable structures, high surface areas and loading capacities, favorable biocompatibility and biodegradability, as well as notable mechanical and thermal stability [8], MOF-based systems have found applications in various fields. These applications encompass catalysis, gas adsorption, purification, separation and storage, nonlinear optics, sensing and detection, and environmental [9,10] and biomedical applications [11].

This systematic review initially examines the recent advancements in employing MOFs for the effective removal of harmful pollutants from wastewater. While previous reviews have discussed the elimination of specific pollutants like heavy metals, dyes, antibiotics, and pesticides, this review aims to fill a knowledge gap by providing a comprehensive assessment of the predominant contaminants in wastewater in a single publication. The primary objective is to present the most up-to-date progress in eliminating pollutants like heavy metals, dyes, pesticides, antibiotics, and endocrine-disrupting compounds from wastewater, while also highlighting the synthesis methods, advantages, and limitations of MOFs. This review particularly emphasizes the latest techniques for synthesizing MOFs, resulting in improved structures and properties that enable faster reactions and higher product yields, as compared to existing adsorptive materials, reducing the reliance on energy-intensive and time-consuming traditional preparation methods. It comprehensively covers the characteristics of MOFs, the interaction mechanisms between MOFs and pollutants, performance evaluations of MOFs for adsorptive pollutant removal, and the key factors influencing their effectiveness, aiming to enhance understanding and provide valuable insights into the feasibility and performance of MOFs as efficient adsorbents for pollutant sequestration in water-based environments.

In addition, the goal of this review is to supply a comprehensive summary of the up-to-date advancements in MOF-based composite materials encompassing their various biomedical applications. These applications include cancer treatment, diabetes therapy and wound healing, and the treatment of brain and neurological disorders, among others. Additionally, recent examples reported in the literature are analyzed in order to clarify the promise of metal–organic frameworks in biomedical applications. Furthermore, the emerging prospects and limitations of using MOFs in biomedicine are presented. In contrast to other excellent reviews in this field, this article not only focuses on notable research conducted on MOFs in the aforementioned areas in recent years, but also offers a comprehensive discussion on the future advancement possibilities and challenges of MOFs in biomedical applications from various perspectives. Summarizing, this review aims also to provide researchers with a thorough understanding of the ongoing status and promising breakthroughs of MOFs in the biomedical field.

## 2. Historical Trajectory of MOF Evolution

Prussian blue, which serves as a representation of a coordination substance, had already been recognized during 18th century. The exploration of materials featuring metal–organic structures was initiated by Tomic [12], who examined the thermal stability of coordinated polymers formed by specific metal ions and ligands. Such primitive materials paved the way for the development of contemporary MOFs. Notably, the research efforts of Hoskins and colleagues played a crucial role in advancing the study of MOFs [13]. Activated carbon and zeolites comprised the prevailing porous materials in last decade of the 20th century [14]. However, their inherent constraints in terms of structural composition hindered their full potential for utilization in biomedical applications, as well as the thorough elimination of pollutants from aquatic environments. Consequently, Yaghi and co-researchers [15] introduced a category of innovative composite materials with enhanced structural integrity and regenerative ability. During their development, these materials were referred to utilizing several terms, such as isoreticular MOFs [16], microporous coordinated polymeric materials [17], porous coordinated networks (PCN) [18], and zeolite-like MOFs [19], as a universally agreed nomenclature had not yet been established.

Currently, extensive research has been conducted on MOFs for various applications, resulting in the synthesis of more than 70.000 MOFs tailored for specific purposes [8]. Significant efforts have been directed towards developing remarkably robust and porous MOFs, including MOF-5, MOF-74 [20,21], etc. Furthermore, considerable notice has been devoted to MIL MOFs, including MIL-53, MIL-96, MIL-100, etc. [22,23]; PCN MOFs like PCN-12, PCN-222, and PCN-515 [24]; zeolitic imidazolate frameworks (ZIFs) (ZIF-7, ZIF-8, and ZIF-67) [25,26]; as well as UiO MOFs, exemplified by UiO-66 and UiO-67 [27]. The aforementioned types of MOFs exhibit exceptional properties attributed to their innovative metal, as well as organic precursors, making them highly suitable for a wide range of industrial applications, including biomedical [28] and environmental [29].

**Figure 1:**
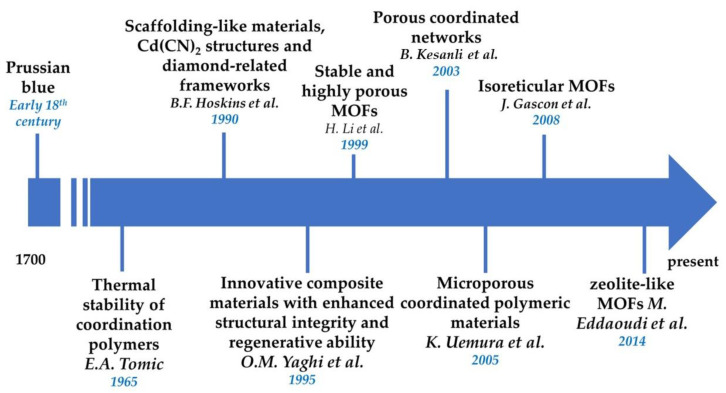
Historical trajectory of the MOFs’ evolution [12,13,15,16,17,18,19,21].

Over the past twenty years, a remarkable surge in the number of research publications exploring various aspects of MOFs has been observed, including their synthesis, properties, and applications. Current investigations are predominantly focused on advancing heterarchical MOFs with enhanced multiplicity and adjustable characteristics [30]. Furthermore, approaches have been devised to functionalize MOFs, enhancing their versatility and scalability. Moreover, researchers are extending the spatial configuration of MOFs from two-dimensional to three-dimensional structures, while retaining their permanent porosity [31,32] (Figure 1).

## 3. Properties and Structural Characteristics of MOFs

**Figure 2:**
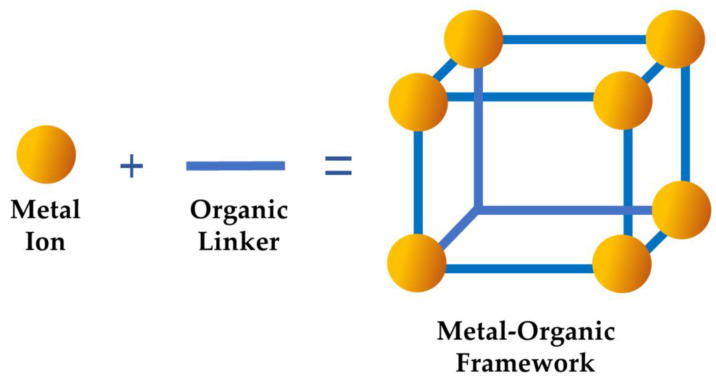
Fundamental MOFs’ structure.

Metal–organic frameworks exhibit a wide range of advantageous characteristics, including enhanced surface area, porosity, and stability under varying conditions [33]. Their porous nature remains intact despite the absence of guest molecules, ensuring that the pore architecture remains unaltered [34]. The creation of pores, which contribute to the metal–organic frameworks’ functionality, is largely influenced by organic linkers. These linkers, when combined with metallic ions, give rise to various MOFs. In the fundamental structure of MOFs, various metal ions (bonded to oxygen atoms, constituting secondary structural units) can be joined through diverse organic linkers, resulting in a wide range of MOF materials (Figure 2). The Cambridge Crystallographic Data Centre has reported the availability of hundreds of distinct secondary building units [35]. Notably, the central atom in the secondary building units can be replaced while maintaining the organic linker, leading to the formation of diverse metal–organic frameworks. The specific class of MOF is defined by the metal precursor and organic linker selection.

**Figure 3:**
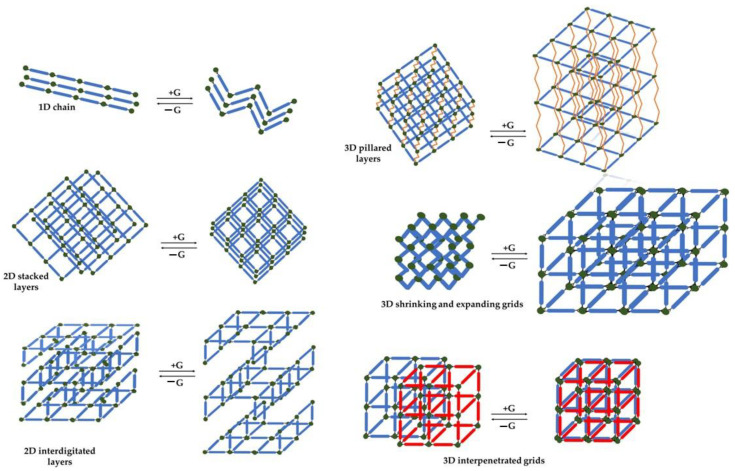
MOF dimensionality, where G stands for the guest molecules, green dots correspond to metal ions, blue lines indicate the organic linkers, yellow lines represent channels and red lines stand for different MOF motifs.

MOFs exhibit a range of crystalline structures with varying topologies and dimensionalities, determined by the metal ion’s geometry and the linker’s binding mode [36]. The dimensional classification of MOFs is based on whether guest molecules are present or absent. Each MOF possesses a unique structural dimensionality, such as 1D, 2D, or 3D (Figure 3). For example, Qiu and co-researchers [37] synthesized Co-MOF-74, a cobalt-based metal–organic framework with a rod-like structural arrangement, to serve as a catalyst in the Fischer–Tropsch procedure. The researchers perceived the rod-like structure of the produced metal–organic frameworks, supported by an increased BET surface area (1025 m^2^/g). Similarly, Strauss and his team [38] synthesized a rod-like Co-MOF-74, validated through SEM analysis. The developed metal–organic framework exhibited a 1D structure with a corresponding hexagonal channel diameter measuring 11 Å.

Two-dimensional (2D) metal–organic frameworks are formed by stacking single layers through edge-to-edge or staggered arrangements, resulting in feeble layer interactions. The stacking pattern is able to be modified through the alteration of the organic linker, playing a role in determining the inclusion of guest molecules within the empty spaces among the layer grid or within the spaces between the layers themselves. Compared to 1D metal–organic frameworks, 2D MOFs possess several advantages, including enhanced electron transferability, a greater number of unveiled catalytic sites, thickness within the nanoscale, as well as improved charge and mass transfer features [39]. Li and his team, in 2021 [40], employed the hydrothermal method to synthesize various 2D polyhalogenated cobalt-based metal–organic frameworks utilizing fluorine, chlorine, and bromine as typical halogens. These synthesized MOFs were evaluated for their electrochemical hydrogen evolution properties. In a related study, also in 2021, Abrori and co-researchers [41] synthesized a cobalt-based MOF-71 featuring a layered structure with sheet packing, as verified through SEM analysis. The prepared metal–organic framework was utilized for electrochemical sensing applications targeting uric acid.

Three-dimensional (3D) metal–organic frameworks are characterized by a distribution of coordination bonds in multiple directions, yielding exceptionally stable and permeable substances. The predominant types of 3D MOFs are pillared layered and grid metal–organic frameworks. Such forms of MOFs are created through robust chemical bonding between organic ligands and metal ions, possessing in their vast majority enhanced surface area, increased pore volume, as well as homogeneous pore size distribution [42]. Moreover, Wang and co-researchers [43] synthesized various Co-based 3D metal–organic frameworks employing diverse linkers to establish a fundamental *gis* topology. The aforementioned metal–organic frameworks were described in terms of their distinct specific surface areas. In a different research, Luo and his team [44] employed a 2D Co-layered double hydroxide as a precursor for the development of a three-dimensional ZIF-67, afterwards modified into 3D hierarchical Co-based polyhedral arrays through thermal treatment. Another method for preparing 3D MOFs is the “pillar-layer” approach, where pillars are introduced into 2D-layered metal–organic frameworks. For instance, Meng and his team [45] incorporated pyromellitic acid pillars into a Zn-based 2D metal–organic framework structure, resulting in a durable 3D MOF that was utilized for the isolation of transition metal ions. A resembling strategy was employed by Han and his team [46] to produce a 3D non-interpenetrated Co-based MOF utilizing N,N’-di-(4-pyridyl)-1,4,5,8-naphthaleneteracarboxydiimide pillars. The corresponding analysis of its structural characteristics unveiled an enhanced specific surface area (755.6 m^2^/g), coupled with a 3D *hex* topology.

Based on the reviewed literature, it is evident that metal–organic frameworks exhibit a wide range of structures, topologies, and pore characteristics. Notably, the size of the pores is primarily determined by the length of the carbon chain within the organic linker, while the introduction of functional groups by the linker influences the chemical attributes and additional functionalities [47]. A fascinating aspect of MOFs is their tunability, which allows for the manipulation of their properties through the selection for initial substances, fabrication procedure, and experimental settings. The chemical and thermal stability of MOFs play a significant role in their suitability for various applications. For instance, evaluating the change in properties of MOFs under different pH conditions using X-ray diffraction tests provides insights into their chemical stability. Thermogravimetric analysis is commonly employed to assess the thermal stability of MOFs [48].

## 4. Synthetic Routes for MOF Development

Various techniques exist for synthesizing MOFs, encompassing both traditional and more recent methods. Traditional approaches include various approaches, such as solvothermal, hydrothermal, mechanochemical, microwave, ultrasonic, as well as ambient temperature stirring. However, up-to-date advancements have introduced novel techniques for producing MOF derivatives with enhanced properties, opening up new possibilities for their applications [49,50]. These innovative methods encompass the electrospinning and carbonization approach, as well as surfactant and deep eutectic solvent-based approaches. Each of these techniques possesses its own advantages and limitations. When it comes to removing contaminants from aqueous solutions, the selection of a specific method depends on the particular requirements of the process, which are discussed in subsequent sections.

### 4.1. Solvothermal/Hydrothermal Approach

**Figure 4:**
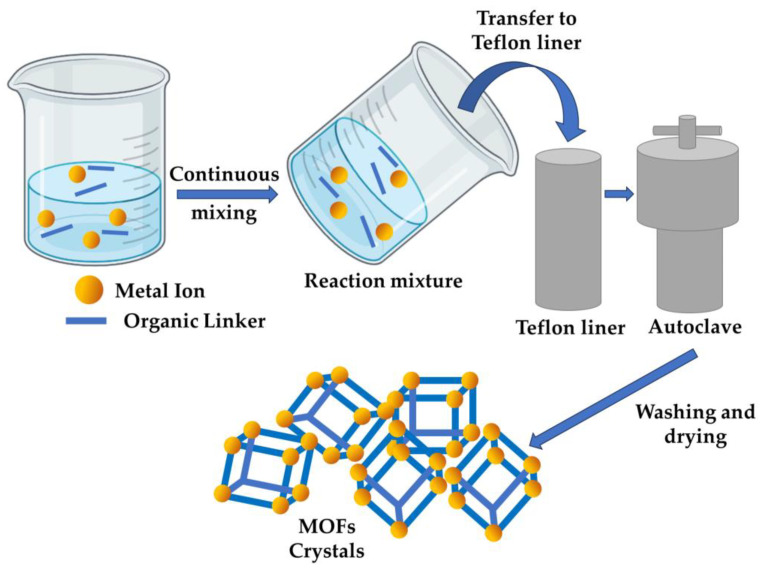
Schematic illustration of the solvothermal/hydrothermal approach for MOF synthesis.

The hydrothermal approach, a variant of the solvothermal technique, employs H_2_O as a solvent [51]. According to this approach, both the precursor (metal ion), as well as the organic ligand, are solubilized within the solvent and consistently blended within a PTFE (polytetrafluoroethylene) liner. The mixture is then subjected to a reaction in an autoclave under predetermined settings regarding temperature, pressure, as well as duration [52]. When ongoing reaction is complete, the end products are cooled to room temperature and subsequently rinsed several times with distilled water to eliminate contaminants, yielding the aforementioned metal–organic frameworks (Figure 4). To obtain pure MOFs, synthesized MOFs undergo further purification, involving cleaning using a proper solvent, such as anhydrous ethanol, subsequently subjected to vacuum drying [53]. The solvents employed in the solvothermal procedure usually consist of exceedingly polar proton donors, characterized by elevated dielectric constants like CH_3_OH, CH_3_CH_2_OH, H_2_O, CH_3_COOH, C_2_H_3_N, DMS, DMF, and C_3_H_6_O [54,55]. An essential aspect of the solvothermal approach entails fine-tuning the reaction parameters, including temperature, duration, solvent selection, and solvent concentration [31]. The selection and concentration of chemical regulators directly impact crystallites’ type and size, which have been instrumental in producing extremely porous, chemically robust materials with unvarying structures in modern advancements [56] (Table 1).

Yaghi and Li [57] were pioneers in utilizing the previously described technique for the synthesis of rectangular-shaped metal–organic frameworks with Cu ions and an aromatic ligand as the organic binding unit. X-ray analysis confirmed the structure of the synthesized metal–organic frameworks, revealing a prolonged framework possessing trigonal planar Cu centers. The solvothermal method offers the advantage of obtaining single crystals, but it also presents limitations such as reduced yield, extended reaction times, as well as increased energy requirements due to the elevated temperatures [52]. To enhance the yield, Hsieh and co-researchers [58] refined the reaction temperature, ligand concentration, and reaction duration, leading to the successful fabrication of exceptionally crystalline Cu metal–organic frameworks. Their properties were directly influenced by these factors, as indicated by XRD studied, which unveiled an association between augmented mobility size and crystallinity of the Cu metal–organic framework. In an effort to address the limitations of Fe-based MOFs, Nguyen and co-researchers [59] employed a modified solvothermal method to synthesize bimetallic MOFs with outstanding properties. The resulting material exhibited a spindle-shaped morphology dominated by micropores and demonstrated 96% removal of rhodamine B under visible light irradiation for 2 h. Additionally, the material exhibited excellent long-lasting reusability with limited decrease in photocatalytic effectiveness after five usage cycles. Then, Denisov and his team [60] solvothermally prepared a novel MOF utilizing a 3D-printed autoclave. Both XRD and elemental studies confirmed the crystallinity and composition of the aforementioned MOF. Nevertheless, they encountered constraints in the method, as the MOF (Zn_4_(BDC)_3_(OAc)_2_(DMF)_4_) formed instead of the intended (Zn_4_O(BDC)_3_), which was associated with alterations in the solvothermal conditions possibly influenced by the compromised integrity of the polypropylene-based autoclave. Zorainy and his team [61] employed a feasible solvothermal approach to fabricate a V_2_O_5_-based MOF (MIL-47 V). Through this approach, they reduced the reaction temperature and time (18% and 72%, respectively), achieving at the same time a chemically and thermally stable MIL-47.

Despite being widely used in the early stages of MOF synthesis, the solvothermal method has limitations such as prolonged reaction times, increased energy requirements, as well as the use of expensive organic solvents. Moreover, this approach might not be appropriate for thermally sensitive starting materials, and there are safety considerations associated with the manipulation of corrosive substances and the development of by-products following the reaction. In the literature, various metal–organic frameworks fabricated using the hydrothermal/solvothermal approach have been distinguished by their enhanced porosity and specific surface area, verifying their potential for environmental and biomedical applications. Analysis of the reviewed research reveals a preference for transition metals and organic ligands. Some studies have also employed a combination of organic ligands. The commonly utilized solvents are extremely polar, such as H_2_O, CH_3_CH_2_OH, CH_3_OH, or a mixture of them, along with DEF (diesel exhaust fluid) and DMF (dimethylformamide). In acidic media, HF (hydrogen fluoride) or HNO_3_ (nitric acid) have been employed as solvents when necessary [62].

**Table 1:** MOF features fabricated using the solvothermal/hydrothermal approach.

Type of MOF	Precursor Materials		Solvent Type	Experimental Parameters	Comments	Ref.
	Organic Ligand	Metal Salt				
MIL-47	C_8_H_6_O_4_	V_2_O_5_	DMF	180 °C 20 h	Chemical and thermal robustness	[61]
UiO-66	C_8_H_6_O_4_	Co_3_O_4_	DMF	120 °C 24 h	Charge separation, as well as visible irradiation adsorption increasement	[63]
SIMOF-4	C_8_H_6_O_6_	Ca(NO_3_)_2_·2H_2_O	C_2_H_6_O/H_2_O	120 °C 72 h	Exceptional electrochemical features	[64]
Cd/Zr MOF	C_8_H_6_O_4_	CdCl_2_	DMF	120 °C 2 h	Enhanced photocatalytic efficiency	[65]
Bi MOF	C_6_H_3_(CO_2_H)_3_	Bi(NO_3_)_3_·5H_2_O	DMF	120 °C 24 h	Presence of microporosity	[59]
Ni/Mn MOF	C_6_H_3_(CO_2_H)_3_	Ni(CH_3_COO)_2_·4H_2_O Mn(CH_3_COO)_2_·4H_2_O	C_2_H_6_O/H_2_O	150 °C 15 h	Exceptional electrochemical features	[66]
La MOF	La(NO_3_)_2_·6H_2_O	H_3_L	DMF	90 °C 72 h	Enhanced sensitivity to amino acids, as well as antibiotics	[67]
Typ MOF	C_15_H_11_N_3_	Ni(NO_3_)_2_·6H_2_O	C_2_H_6_O/H_2_O	160 °C 120 h	Caffeine adsorption ability	[68]

### 4.2. Microwave Approach

The synthesis of MOFs can be achieved using microwave-facilitated methods, which utilize microwave energy within the range of frequencies 300–300,000 MHz. This approach offers an energy-effective and environmentally friendly path for MOF fabrication. Unlike traditional heating methods, microwave-facilitated synthesis relies on the interplay among the moving charges in the polar solution and the microwave radiation to provide the required heat. This method ensures a consistent temperature rise throughout the reaction, promoting crystal development in MOF fabrication. The unique thermal features of the microwave approach enable enhanced regulation of crystal size and structure, reduced reaction durations, and increased production efficiency, selectivity, and purity. Consequently, the microwave approach has emerged as a highly effective approach for synthesizing MOFs. 

Numerous studies conducted over the past decade have explored the microwave method for MOF synthesis (Table 2). For instance, Moustafa and co-researchers [69] successfully produced a zeolitic metal–organic framework (ZIF-8) within a short period of 15 min using a microwave-assisted solvothermal process. XRD analysis confirmed the formation of ZIF-8. Correspondingly, Dang and his team [70] achieved effective fabrication of Zr-UiO-66 and Hf-UiO-66 in just a few minutes. The resulting metal–organic frameworks exhibited decreased particle sizes, similar porosity to the original framework, as well as increased removal effectiveness for curcumin in H_2_O solutions. Microwave synthesis was also employed by Albuquerque and Herman [71] to produce nickel-based MOF-74 using C_7_H_6_O_2_ as a chemical modulator. The prepared metal–organic frameworks exhibited enhanced specific surface area, crystallinity, and narrow particle size distribution. Furthermore, Bazzi and his team [72] successfully synthesized ZIF-8 from ZnO utilizing a mixture of solvents via the microwave method, offering a novel approach to synthesizing MOFs from inorganic metal oxides. The resulting ZIF-8 displayed desirable morphologies and optimal adsorptive capacity for phosphate removal from solutions. In addition, Gaikwad and co-researchers [73] utilized the microwave approach to synthesize a novel Zn-based MOF called UTSA-16, in which Co was replaced with Zn. The metal–organic frameworks exhibited significantly enhanced stability when exposed to acid gases and moisture, surpassing cobalt-containing MOFs in terms of CO_2_/N_2_ selectivity. Furthermore, UTSA-16 demonstrated remarkable CO_2_ capture capabilities. A novel approach combining microwave irradiation with perturbation-assisted nanofusion was employed by Laha and his team [74] to fabricate metal–organic frameworks with hierarchical porosity. Through the fusion of microporous MOFs using various solvents, mesopores were generated within a shorter reaction time. The resulting MOFs possessed the ability to encapsulate enlarged biomolecules like vitamin B12. Zorainy and his team [75] utilized a microwave-assisted solvothermal process to synthesize MIL-88B, a 3D Fe-based MOF, from a trivalent iron salt and C_8_H_6_O_4_. The synthesized MIL-88B exhibited significant Fe and O contents of 24% and 29%, respectively, highlighting its suitability as an oxygen-rich catalyst. TGA and DSC tests confirmed the exceptional catalytic ability and thermal stability of the produced metal–organic frameworks. Wei et al. [76] employed a combination of microwave irradiation and ball milling to synthesize Sm MOFs, which displayed notable adsorption capacity for Congo red dye in solution. Utilizing 50 mg of Sm MOFs, an adsorption capacity equal to 396.8 mg/g was achieved for an initial Congo red dye concentration of 80 mg/L. Moreover, Nguyen and his team [77] utilized a combined microwave and solvothermal approach to rapidly synthesize Zr-based MOFs for the removal of toxic organic dyes. The synthesized metal–organic frameworks exhibited exceptional adsorptive capacity for methyl orange and methylene blue, demonstrating enhanced reusability over multiple adsorption cycles. 

It is worth noting that these studies consistently reported decreased reaction times and enhanced product yield, highlighting the efficiency of this synthesis route. Transition metal salts, particularly zirconium and nickel, were commonly used as precursors, with C_8_H_6_O_4_ and DMF being the prevailing organic ligand and solvent, respectively. While the microwave method offers advantages such as minimal side product formation, it is important to acknowledge some reported drawbacks, including challenges in result reproducibility attributed to variations in microwave equipment [78].

**Table 2:** MOF features fabricated using the microwave approach.

Type of MOF	Precursor Materials		Solvent Type	Experimental Parameters	Comments	Ref.
	Organic Ligand	Metal Salt				
Zr-UiO-66 Hf-UiO-66	C_8_H_6_O_4_	ZrCl_4_ HfCl_4_	DMF	110 °C 3 min	Exceptional curcumin removal efficiency	[70]
UTSA-16	C_6_H_8_O_7_	Zn(CH_3_COO)_2_·2H_2_O	C_2_H_6_O/H_2_O	90 °C 240 min	Increased robustness and selectivity Exceptional CO_2_ capture efficiency	[73]
Ni-MOF-74	DHBDC	Ni(NO_3_)_2_·6H_2_O	DMSO DMF	100 °C 40 min	Adjustable porosity	[74]
MIL-88B	C_8_H_6_O_4_	FeCl_3_·6H_2_O NiCl_2_·6H_2_O	DMF	100 °C 60 min	Exceptional photocatalytic attributes	[77]
Cd/Zr MOF	C_8_H_6_O_4_	ZrCl_4_ CdCl_2_	DMF	120 °C 30 min	Enhanced photocatalytic efficiency	[65]
Al-MIL-53	C_8_H_6_O_4_	AlCl_3_·6H_2_O	DMF	220 °C 2 min	Exceptional furfural separation ability	[79]
Zr MOF	CH_2_O_2_	ZrOCl_2_·8H_2_O	DMF	100 °C 60 min	Efficiency in gas separation applications	[80]
UiO-66	C_8_H_6_O_4_	ZrCl_4_	DMF	120 °C 30 min	Efficiency in sensing applications	[81]

### 4.3. Sonochemical Approach

**Figure 5:**
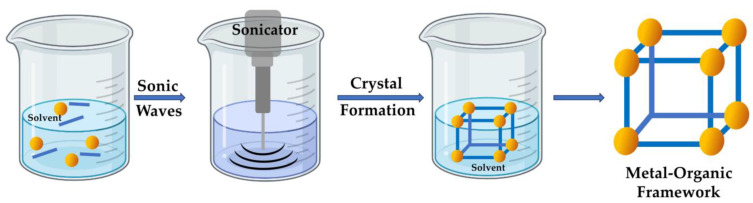
Schematic illustration of the sonochemical approach to MOF synthesis, where the yellow color is used for representing the metal ions and blue color is used for indicating the organic linkers.

The sonochemical approach to fabricating metal–organic frameworks utilizes ultrasound radiation (20–10,000 kHz) (Figure 5). Similar to the microwave method, it offers a rapid, straightforward, low-temperature, and effective approach for developing MOFs with increased specific surface areas [52]. This approach relies on acoustic cavitation, where bubbles form and collapse in the solution, resulting in localized hotspots [82]. This process generates pressures of up to 1000 atm and temperatures exceeding 5000 °C in the cavitation zone, offering the necessary energy for the reactions’ conduction. Compared to hydrothermal and solvothermal approaches, the increased temperatures in sonochemical approaches promote swift chemical reactions, allowing for immediate crystal formation through uniform nucleation centers and decreasing both crystallization time and crystallite size.

Recent researches have extensively explored the sonochemical approach as an effective means of fabricating metal–organic frameworks due to its capability to regulate crystal size, phase selectivity, as well as reaction time (Table 3). Hayati and co-researchers [83] employed an ultrasound-assisted process to prepare an Ag-based metal–organic framework using AgNO_3_ and C_7_H_6_O_3_ dissolved in CH_3_OH (solvent). They investigated the effects of reaction time and temperature, ultrasonic power, and reactant concentration, ultimately obtaining a Ag MOF with exceptional catalytic efficiency under specific conditions: a temperature of 70 °C, a reaction time of 60 min, a reactant concentration of 0.1 M, and an ultrasound power of 60 W. SEM studies verified the presence of micro- and nanorod shapes in the produced metal–organic frameworks. The synthesized Ag MOF exhibited high removal percentages of C_8_H_6_Cl_2_O_3_ and C_9_H_9_ClO_3_ from solution under solar irradiation (96% and 98%, respectively). Zr-based MOF-525 and MOF-545 were also fabricated using a sonochemical procedure, utilizing ZrCl_4_ 8H_2_O as the metal precursor and tetrakis (4-carboxyphenyl) porphyrin as the organic linker [84]. Subsequent SEM analysis indicated that MOF-525 exhibited a cubic shape with enhanced specific surface area (2511 m^2^/g) and pore volume (1.21 cm^3^/g). The size of the particles ranged from 0.2 to 0.3 μm. On the other hand, MOF-545 had a needle-like shape with decreased specific surface area and particles, with sizes ranging from 0.8 to 1.2 μm. The synthesized metal–organic frameworks demonstrated improved hydrolysis of C_8_H_10_NO_5_P and enhanced adsorption efficiency for bisphenol-A compared to conventionally prepared MOF materials.

In a recent investigation, Huang and colleagues [85] employed a sonochemical method to synthesize a sheet-like Ln MOF that significantly enhanced the chemiluminescence emission of the NaIO_4_-H_2_O_2_ system. The fabricated a Ln MOF exhibited a substantial increase in chemiluminescence, reaching up to 67 times higher intensity.

In another study by Fouad and his team [86], a novel Co-based metal–organic framework was developed for the detection of Cr (III) ions in water. The characterization results demonstrated the high sensitivity and selectivity of the Co MOF towards Cr(III). The hydrophobic material possessed a specific surface area of 270.2 m^2^/g, pore diameter equal to 5.05 nm, as well as an average particle size from 45 to 73 nm. The aforementioned metal–organic framework presents a promising alternative for accurate determination of Cr(III) in real water samples. 

This approach has been collaboratively coupled with conventional synthetic techniques to enhance their functionality. More specifically, Abdolalian and co-researchers [87] integrated sonochemical and solvothermal approaches to fabricate TMU-42, which constitutes a functionalized metal–organic framework. The resulting nanorod-shaped metal–organic frameworks exhibited exceptional catalytic effectiveness in the Henry and Knoevenagel reactions and maintained their structure and catalytic efficiency after four usage cycles. In addition, Karbalaee and Tadjarodi [88] developed a Cd MOF from Cd(NO_3_)_2_ 4H_2_O and 5-aminoisophthalic acid through a similar approach, achieving a faster reaction time (1 h) compared to the conventional solvothermal method that required 48 h. The Cd MOF served as a precursor for the production of CdSO_4_ nanoparticles through thermal treatment at 600 °C. In their research on antimicrobial encapsulation and caffeic acid loading, Shen and his team [89] developed cyclodextrin-based metal–organic frameworks (U-CD-MOF). The utilization of ultrasonic energy significantly reduced the time of the reaction to just a few minutes. An analysis of sensitivity revealed that the duration of the reaction, as well as the temperature and power of the ultrasound had a notable impact on the size and structure of the MOFs. Through optimization, a cubic-shaped U-CD-MOF was achieved, exhibiting a loading capacity for caffeic acid of 19.63%, which represented a 100% improvement compared to pristine cyclodextrin. The sonochemical synthesis method offers the advantage of controlling the linkages within the framework by manipulating the ultrasound power level.

Upon critical examination of the literature, it was evident that the sonochemical approach was commonly employed to enhance traditional synthesis methods. In numerous instances, the incorporation of the sonochemical method resulted in improved performance. Additionally, it was observed that reaction conditions, including ultrasound power level, temperature, time, and reactant concentration, influenced the process effectiveness and MOF properties. Notably, ultrasound power level played a significant role in determining the framework’s linkages. Typically, the sonochemical method yielded MOFs with smaller particle sizes and reduced crystallization times under mild reaction conditions, distinguishing it from typical techniques like hydrothermal or solvothermal approaches.

**Table 3:** MOF features fabricated using the sonochemical approach.

Type of MOF	Precursor Materials		Solvent Type	Experimental Parameters	Comments	Ref.
	Organic Ligand	Metal Salt				
U-CD-MOF	Cyclodextrin	KOH	CH_3_OH	20 kHz 10 min 60 °C	Efficiency in caffeic acid loading	[89]
TMU-34	3,6-di(4-pyridyl)-1,4-dihydro-1,2,4,5-tetrazine	Zn(CH_3_COO)_2_·2H_2_O	DMF	40 kHz 160 min 120 °C	Efficiency in sensing applications	[90]
MOF-525	Tetrakis (4-carboxyphenyl) porphyrin	ZrCl_4_·8H_2_O	DMF	20 kHz 150 min 80 °C	Enhanced surface area and pore volume	[91]
MOF-545	Tetrakis (4-carboxyphenyl) porphyrin	ZrCl_4_·8H_2_O	DMF	20 kHz 30 min 80 °C	Enhanced surface area and pore volume	[91]
Co MOF	C_9_H_6_O_6_	Co(CH_3_CO_2_)_2_·4H_2_O	Distilled water	40 kHz 30 min 25 °C	Increased Congo red dye removal effectiveness	[92]

### 4.4. Mechanochemical Approach

**Figure 6:**
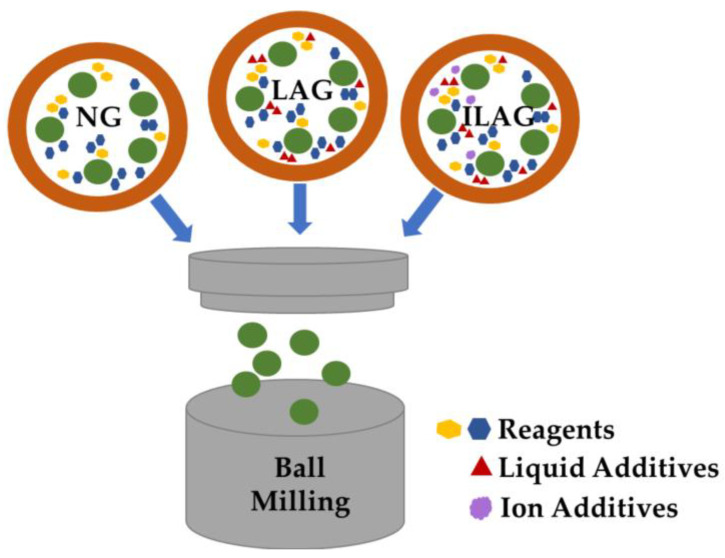
Schematic illustration of the various mechanochemical approaches for MOF synthesis, where in green are illustrated the balls utilized during the ball milling synthetic procedure.

In this approach, the chemical conversion of precursor materials into MOFs is achieved by harnessing the mechanical energy generated through grinding (Figure 6). As explained by Titi et al. [93], this process can be divided into three stages. Initially, the mechanical action, such as shearing, grinding, or milling, breaks down the bond structure of the precursor material, resulting in a finely powdered form with enhanced crystallinity and surface area. Subsequently, under van der Waals forces, the powder particles aggregate and further agglomerate to develop MOF crystals through a mechanochemical reaction. Ball milling is a commonly employed technique for this purpose, requiring increased impact energy to trigger the desired chemical transformations [94]. While traditional methods involved simple mortars and pestles, specialized ball mills are now used to provide consistent conditions and high impact energy for mechanochemical reactions. 

The mechanochemical approach can be categorized into three types: (a) neat grinding (NG), which is characterized by the absence of solvents; (b) liquid-assisted grinding (LAG) that utilizes a small amount of solvent; and (c) ion- and liquid-assisted grinding (ILAG). Neat grinding allows for the use of insoluble metal precursors that are challenging to dissolve in traditional solvents for MOF production, presenting a safer and more environmentally friendly approach. It has been noted that the inclusion of a minute quantity of water in the precursors augments the mobility of the organic linker and metal ions, thereby facilitating the establishment of coordination bonds throughout the reaction [94]. This principle underlies the liquid-assisted grinding mechanochemical approach that employs small quantities of solvents to improve the process efficiency. Compared to neat grinding, liquid-assisted grinding offers faster reaction times and yields more crystalline materials. The ion- and liquid-assisted grinding method incorporates both salts and solvents as additives, enhancing the dissolution of solid precursors and overall process effectiveness.

Table 4 presents a summary of research studies that have utilized the mechanochemical approach to fabricating metal–organic frameworks. From the literature examined, it is evident that the mechanochemical method offers an environmentally friendly approach to green MOF synthesis. In comparison to conventional solvent-based approaches, this approach can be conducted at room temperature and enables the rapid formation of crystals within minutes, a significant improvement over the hour-long process of solvent-based approaches (Table 5). Moreover, the utilization of a solvent-free medium eradicates the need for managing liquid waste after the reaction. In general, metal oxides, particularly ZnO, have been employed as metal precursors instead of metal salts, resulting in the production of H_2_O as the sole byproduct. A few studies incorporated a small amount of solvent to aid the grinding process, leading to accelerated reactions. Ion- and solvent-assisted grinding was employed in certain instances, yielding pillared layered MOFs. Despite the evident benefits of the mechanochemical procedure, Table 5 indicates that it possibly gives rise to amorphous regions in the produced metal–organic frameworks. Furthermore, achieving reproducible results necessitates the use of specialized mills and grinders, making precise process control challenging. There is also a possibility of introducing impurities.

### 4.5. Ambient Temperature Stirring Approach

**Figure 7:**
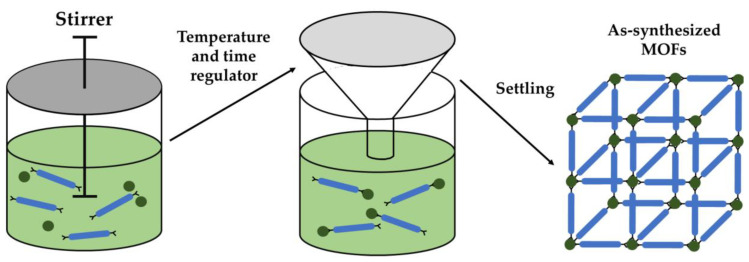
Schematic illustration of the ambient temperature stirring approach for MOF synthesis, where green color is used for representing the metal ions and blue colored lines stand for the organic linkers.

In the stirring approach, the metal precursor, organic linker, and other solvents are combined and continuously agitated at a definite temperature for a defined period. Subsequently, the resulting reaction product is filtered, and the residual solvent is evaporated (Figure 7). Zhao and his team [103] employed this approach to fabricate ultrathin 2D ZIF-67-NS for efficient removal of As(III) from water. ZIF-67-NS demonstrated exceptional water stability under alkaline conditions and exhibited an increased adsorption capacity of As(III) due to its dimensional characteristics. Furthermore, Tong and his team [104] prepared biomolecular metal–organic frameworks (BZIF-8) by stirring a mixture of 2-methylimidazole and Zn(CH_3_COO)_2_ 2H_2_O at ambient temperature, yielding a multiphased BZIF-8 structure with both amorphous and crystalline phases. The crystal growth mechanism was ascribed to electrostatic interactions between the precursors, and the presence of the amorphous phase led to a large pore opening, offering potential catalytic and pore transport properties. Sui and his team [105] studied a boronation process of Ni-Co MOFs at ambient temperature and a subsequent phosphating in order to activate the metal compounds. The resulting material presented outstanding electrochemical features, including an increased capacitance of 1578 F/g, and demonstrated excellent stability with 87% retention after 5000 charging–discharging cycles.

The fabrication of metal–organic frameworks at room temperature is highly favorable and has garnered increased interest in the field of sustainable chemistry. The research studies compiled in Table 6 provide an overview of the ambient temperature synthesis method employed for metal–organic frameworks. This approach offers simplicity and ease of implementation since it does not necessitate enhanced temperature or pressure conditions. By manipulating the reaction conditions, for example, adjusting the temperature or carefully evaporating the solvent, it is possible to control the reactant concentration, surpassing the nucleation threshold and promoting crystal growth from a clear solution. Moreover, metal–organic frameworks synthesized at ambient temperature tend to exhibit chemical and thermal stability. However, a notable drawback of this approach is the relatively lower purity of the developed product, primarily due to partial removal of the solvent.

### 4.6. Electrospinning Approach

Currently, there is significant emphasis on altering the structures of MOFs at the meso- and macroscale to accommodate various applications like membranes, coatings, and advanced device architectures [112]. The use of inorganic materials as binders for structuring MOFs is limited due to the requirement for heat treatment. To address this limitation, polymers have emerged as a promising approach to achieving MOF structuring. The incorporation of polymers with metal–organic frameworks enhances chemical stability, as well as mechanical flexibility. However, a significant struggle in integrating metal–organic frameworks into polymer matrices is the inferior dispersion and probable incompatibility resulting from the contrasting nature of the two materials [113]. Electrospinning has emerged as a reliable technique for structuring metal–organic frameworks into hybrid materials with additional functionalities. The products resulting during electrospinning exhibit sizes ranging from tens of nanometers to a few micrometers, taking the pattern of nonwoven yarns and mats [52]. Throughout the electrospinning process, a polymeric solution characterized by increased viscosity is extruded in a columnar shape through a needle under the influence of high voltage. The discharged product solidifies in the electrically charged jet on a collector [114]. Electrospun metal–organic framework derivatives possess a three-dimensional nanoscale porous structure, offering distinctive features for various applications. This approach is usually employed for fabricating composite nanofibers containing MOFs and polymers. In this approach, a slurry containing MOFs is combined with a polymeric solution and directly electrospun to form a composite nanofiber, embedding the MOFs within the polymer matrix. The electrospinning procedure can be refined by considering factors such as solvent and polymer selection and the electrospinning conditions including the needle–collector distance, voltage, and slurry viscosity, depending on the target metal–organic framework. Table 7 presents an overview of investigations that have utilized the electrospinning technique in the synthesis of MOFs.

### 4.7. Carbonization Approach

The carbonization approach is utilized for creating MOF derivatives through their subjection to high-temperature treatments. This process involves calcination and pyrolysis of metal–organic frameworks at elevated temperatures, resulting in nanoscale carbon materials with distinct properties [49,121]. By controlling the calcination temperature and duration, it becomes possible to acquire metal–organic framework-derived carbon materials exhibiting increased surface areas and exceptional adsorptive ability. Additionally, the features of the resulting carbon can be adjusted via modifying the composition of the metal–organic frameworks’ mixture through the addition of other organic substances and metals [122]. The production of porous carbon materials from MOFs can be achieved via three primary approaches: (a) direct carbonization of metal–organic framework precursors, (b) co-carbonization of metal–organic frameworks with carbon-containing materials, and (c) post-processing of metal–organic frameworks. Each of the aforementioned fabrication approaches influences the characteristics of the final carbon material. MOF-derived carbon materials exhibit superior physical and chemical properties and possess well-organized porous structures, making them highly attractive to researchers compared to commercially available activated carbon.

A novel approach was developed by Jian et al. [123] to produce three distinct types of porous Co_3_O_4_ materials through the direct pyrolysis of a ZIF-67 MOF material at different temperatures: 350, 450, and 550 °C. The resulting Co_3_O_4_-350, Co_3_O_4_-450, and Co_3_O_4_-550 exhibited hollow, yolk–shell, and hierarchical porous cubic structures, characterized by ultrafine building blocks. The described porous Co_3_O_4_ materials showed great potential as anode materials for lithium-ion batteries (LIBs). Between the aforementioned anode materials, the Co_3_O_4_-450 nanocubes demonstrated the most promising performance with a specific efficiency of 1148 mA·h/g and a rate efficiency equal to 317 mA·h/g at 10 A/g after 200 repeating cycles. The observed lithiation/delithiation behavior could possibly be ascribed to the hierarchical porous structure and core–shell architecture, as well as to the presence of hollow structures. The Co_3_O_4_ nanocubes and the fabrication approach presented within that study are expected to pave the way for the development of optimized anode materials for LIBs, ultimately leading to significantly increased energy density in future battery technologies.

Ma and his team [124] conducted a study where a ZIF-67 MOF material was directly developed on wood in order to create self-supported ZIF-67/wood composite structures. Subsequently, they subjected these structures to carbonization at various temperatures, aiming to produce 3D porous carbon skeleton/magnetic composites, denoted as Co/C@WC. The carbon skeleton, formed from the carbonized wood, established a three-dimensional conductive network, while magnetic nanoparticles of Co/C core–shell configuration were homogeneously embedded within this carbon skeleton. The proposed integration significantly enhanced both the electrical conductivity and magnetism of the resulting composite. At a carbonization temperature equal to 1000 °C, the Co/C@WC composite exhibited exceptional conductivity, measuring 3247 S/m, as well as an average EMI SE_T_ (electromagnetic interference shielding effectiveness) of approximately 43.2 dB within the frequency range of 8.2–12.4 GHz. Additionally, the Co/C@WC composites displayed favorable attributes, such as sound insulation, temperature resistance, and robust mechanical performance, rendering them suitable candidates for versatile shielding materials, especially under harsh conditions.

Also, Ma et al. [125], in another study, presented a straightforward approach for producing an ultrathin conductive carbonized wood film with a dense layered network structure. Through compression and carbonization pretreatment, the wood-based film’s compacted lamellated structure facilitated electron transport, significantly enhancing its electrical conductivity, measuring 58 S/cm. At a thickness of 140 μm, the pure carbonized wood film exhibited a remarkably high specific shielding effectiveness per unit weight (SSE/t) of 9861.41 dB·cm^2^/g, surpassing other reported wood-based materials for electromagnetic interference shielding. In addition, the wood-based film’s densified layered framework effectively generated numerous electromagnetic wave reflections. Moreover, by introducing dodecahedron ZIF-8 crystals through in situ growth on the carbonized wood film’s surface, a CWF/ZIF-8 composite was effectively synthesized, exhibiting an impressive EMI shielding effectiveness of up to 46 dB at X-band. The aforementioned composite formed a well-connected microcurrent network possessing abundant polarization groups, further leading to excellent electromagnetic performance. Furthermore, its dense layered structure presented enhanced conductivity, enabling multiple scattering and reflection of electromagnetic waves. Additionally, the layered porous wood-based film demonstrated exceptional Joule heating performance due to its increased electrical conductivity. The aforementioned facile synthetic route presented increased promise towards the advancement of wood’s practical utilization in electromagnetic interference shielding, thereby promoting the replacement of conventional nonrenewable and costly materials with novel wood biomass.

In general, the characteristics of carbon materials derived from MOFs are influenced by the type and composition of the precursors, as well as the conditions used during calcination (Table 8). Furthermore, it has been demonstrated that by manipulating these variables, it is possible to adjust the pore structure and volume, shape, and surface area of the resulting carbon materials [122].

### 4.8. Electrochemical Approach

**Figure 8:**
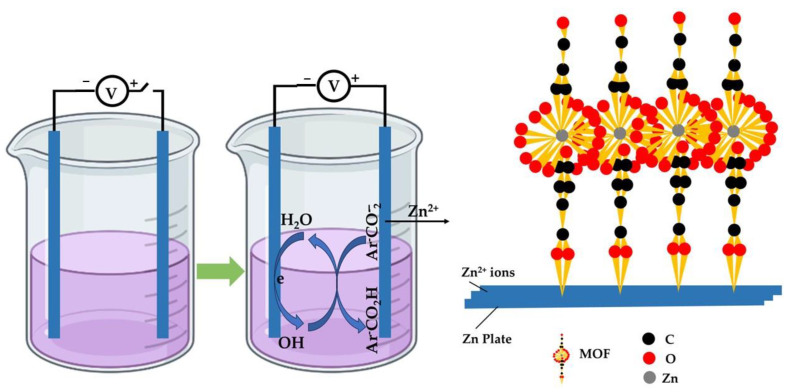
Schematic illustration of the electrochemical approach for MOF synthesis.

This technique has evolved as a continuous method for synthesizing metal–organic frameworks (Figure 8). In comparison to other batch synthetic approaches, the electrochemical approach yields metal–organic frameworks with an enhanced concentration of solid material [78]. Electrochemical approaches can be categorized into various types, including cathodic or anodic deposition and galvanic or electrophoretic displacement. In cathodic deposition, metal–organic frameworks are formed at the cathode through the coordination of metal ions in the solution with deprotonated ligands produced by cathodic reactions [134]. Conversely, in anodic deposition, metal–organic frameworks are produced at the anode by the coordination of metal ions resulting from the anode’s oxidation and the presence of ligands in the electrolyte [135]. Galvanic displacement capitalizes on the disparity in reduction potential between the metal and its substrate, enabling one metal to undergo reduction on the substrate while the substrate experiences oxidation. The resulting metal ions then coordinate with the organic ligand to develop metal–organic frameworks. The electrochemical approach provides various benefits, such as convenient operation and scalability, enhanced crystal growth rate, and gentle reaction conditions. This technique utilizes a battery setup comprising anode and cathode plates submerged in an electrolytic solution containing a metal salt precursor and organic ligand. Solid supports such as FTO (fluorine-doped tin oxide), metal mesh, and ITO (indium tin oxide) glass are commonly used for deposition in this method. The choice of solvent, temperature, and current–voltage density could impact the yield and attributes of the synthesized metal–organic frameworks during the electrochemical procedure.

The reviewed studies highlighted several notable benefits, including the simplicity and rapidity of the synthesis process and the energy efficiency resulting from the mild reaction conditions. Among the frequently employed electrolyte systems, MTBS emerged as the most commonly used. Other commonly utilized electrolytes encompass C_8_H_6_O_4_, C_8_H_15_N_2_Cl, C_14_H_33_NO_4_S, and C_16_H_36_BF_4_N. However, a key drawback of the electrochemical approach is the requirement to dissolve organic ligands in solvents that may present decreased solubility. Additionally, the precise mechanisms underlying cathodic reactions remain incompletely understood, indicating the need for further research in this emerging field of electrochemical MOF synthesis. Each of the different synthetic procedures possesses unique features, benefits, and drawbacks, ultimately influencing the properties of the resulting MOFs. Table 9 provides a summary of these distinct synthesis routes, along with their respective assets and limitations.

## 5. Environmental Applications of MOFs in Wastewater Treatment

Recent data indicate that the global population experienced significant growth from 6.4 to 7.7 billion between 2003 and 2019, and it is projected to exceed 10 billion by 2050 [136,137]. Unfortunately, approximately one third of the world’s total population lacks admission to safe drinking water, which is attributed to the discharge of unprocessed liquid waste into the environment [138]. These untreated effluents contain various harmful contaminants, including toxic inorganic, organic, and biological substances, leading to severe water pollution [8,139]. Notable examples of these contaminants constitute heavy metals, dyes, detergents, pesticides, endocrine-disrupting compounds, antibiotics, as well as pharmaceuticals that have garnered considerable attention from regulators and researchers alike [140]. The accumulation of these pollutants in the environment above acceptable levels imposes significant hazards to both the environment and human wellbeing. Consequently, remediation of contaminated water comprises a pressing global issue [141]. 

**Figure 9:**
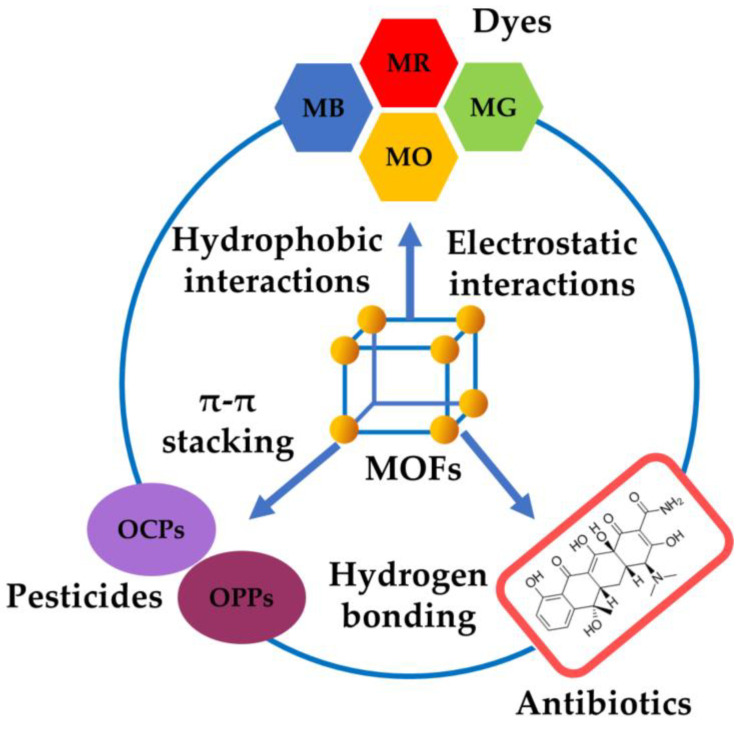
Pollutants’ adsorptive elimination mechanisms utilizing MOFs.

Numerous techniques have been developed for pollutant removal from wastewater, including filtration [142], chemical precipitation [143], coagulation [144], electrochemical treatment [145], membrane separation [146], chemical oxidation [147], photocatalysis [148], ozonation [149], and adsorption [150]. Among these methods, adsorption has garnered considerable interest due to its inherent advantages. Unlike other techniques, adsorption avoids drawbacks like increased initial and operational costs, lengthy treatment durations, process complexity, and space requirements [140]. Adsorption is widely favored due to its enhanced efficiency, feasibility, low energy consumption, versatility, and reusability [151]. Conventional adsorbents utilized for wastewater treatment include clays [152], zeolites [153], ion exchange resins [154], carbon nanotubes [155], silica [156], and activated carbon [157]. Nevertheless, such adsorbents have limitations such as decreased adsorption efficiency, limited reusability, as well as sludge production after treatment [158]. Particularly, metal oxide-based adsorbents exhibit inadequate selectivity and slow adsorption kinetics, while activated carbon is constrained by increased purchase costs, low specific surface area and porosity, as well as diminishing adsorption efficiency over time. Similarly, resins are characterized by confined recovery and reusability potential [159]. Therefore, the latest research endeavors have focused on creating innovative and exceptionally effective adsorbents that can surpass the constraints of conventional alternatives while fulfilling the requirements of environmental sustainability and economic viability. Metal–organic frameworks are an example of such promising adsorbents (Figure 9).

The utilization of metal–organic frameworks in environmental remediation, particularly for the removal of pollutants through adsorption in wastewater, is widespread. This could be attributed to the ease of MOF synthesis and their increased adsorption and regeneration ability, making them appropriate adsorbents for wastewater pollutant removal [160]. Additionally, MOFs possess photocatalytic abilities due to their photoresponsive nature, allowing them to absorb light through their metal centers or organic linkers. This characteristic renders metal–organic frameworks highly effective for wastewater treatment utilizing advanced oxidation processes [50,161]. Notably, metal–organic frameworks have been found to outperform conventional photocatalysts like zinc oxide and titanium dioxide, which face challenges in posttreatment, photocorrosion, as well as limited photocurrent quantum yield deriving from inadequate solar utilization effectiveness [140]. Moreover, the adsorptive properties of MOFs can be enhanced through functionalization [162,163,164], making them optimal materials for pollutant removal from wastewater via adsorption and photocatalysis mechanisms.

### 5.1. Adsorptive Removal of Heavy Metals

Heavy metals encompass metals such as lead (Pb), mercury (Hg), cadmium (Cd), zinc (Zn), chromium (Cr), arsenic (As), nickel (Ni), iron (Fe), and copper (Cu), which have higher atomic numbers and densities than 5 and 20 g/cm^3^, respectively [165]. Human-induced activities and chemical processes are recognized as provenances of heavy metal ions in soil and H_2_O. Inherently, heavy metals, as ions, can readily enter the food chain and have adverse effects on living organisms. Their accumulation in the body can lead to illnesses like cancer given their stability and toxicity [166]. Moreover, the release of heavy metals into water bodies poses threats to both human health and the ecosystem [167]. Specifically, exposure to Pb can have significant negative health impacts on humans and animals. The literature suggests that lead in solution hampers Ca function and protein interactions, underscoring the importance of its removal from aqueous solutions for general human welfare [168]. Similarly, Hg is extremely noxious to human health and widely found in natural sources. Concentrated mercury damages cells, acts as an enzymatic inhibitor, and disrupts the nervous and immune systems [169]. Thus, there is critical demand for efficiently sequestering heavy metal ions from soil and water sources [170].

Traditional methods for eliminating heavy metal ions from water include coagulation, membrane separation, nanofiltration, ultrafiltration, reverse osmosis, flocculation, chemical precipitation, electrochemical removal, as well as ion exchange [171]. The as-mentioned approaches have shown success in wastewater treatment for heavy metal removal. Nonetheless, their commercialization is limited because of their inefficiency for mass manufacturing applications, predominantly attributed to increased operational and maintenance costs. Additionally, a few of these strategies generate sludge during incomplete heavy metal removal [172]. Consequently, there is a significant call to develop a financially viable and efficient method for heavy metal removal to address these limitations.

In comparison to alternative approaches of removing heavy metal ions, adsorption offers potential financial viability, high removal efficiency, ease of implementation, and scalability to industrial systems [173]. The adsorption process relies on adsorbents derived from easily accessible and affordable materials such as biomass, clay, agricultural waste, as well as industrial waste [174]. The effectiveness of adsorbents in the adsorption process is influenced by their nature and composition. Thus, there is a need for cost-effective adsorbent materials with enhanced adsorption capacities that have attracted the attention of researchers. While numerous porous materials can be identified as potential adsorbents, a great number of them possesses limited selectivity and low adsorption efficiencies, while others lack recyclability. To enhance adsorption performance, researchers have focused on the design and synthesis of metal–organic frameworks characterized by exceptional and desirable structural features in relation to traditional adsorbents [172].

This section provides an overview of the use of several metal–organic frameworks for heavy metal removal. Multiple studies have described the retention of metals such as Pb, Cr, Cd, Cu, Hg, As, U, and Ni from aqueous solutions [175,176,177,178,179,180,181]. The removal of heavy metals from water bodies is crucial for the preservation of the environment and researchers have investigated the efficacy of different metal–organic frameworks for such purpose. Specifically, the following section focuses on the removal of Fe, Pd, Hg, As, Cd, and Cu ions from aqueous solutions using MOFs.

Thiol-functionalized Zr MOFs (UiO-66-Cl and UiO-66-S) fabricated via the solvothermal approach were determined to effectively remove iron from water [182]. UiO-66-S exhibited an adsorption efficiency of 481 mg/g for Fe^3+^, while UiO-66-Cl presented an efficiency equal to 279 mg/g. The presence of increased S density in the MOFs contributed to their selectivity towards Fe^3+^ due to the interactional synergy between iron ions and unsaturated sulfur-based functional groups, indicating the potential for H_2_O purification. The adsorption kinetics, represented by the pseudo-second-order equation, revealed higher and faster adsorption rates for UiO-66-S with adsorption rate constants of 1.07 g/mg·h compared to 0.124 g/mg·h for UiO-66-Cl. Furthermore, UiO-66-S demonstrated excellent reusability with a removal efficiency of 89% after six cycles.

Functionalizing metal–organic frameworks with amino acids can increase their effectiveness in adsorbing Pb^2+^ ions. For example, researchers adjusted the NH_2_ group concentration of amino acid-functionalized Al-MIL-53 to improve its capacity for adsorbing Pb^2+^ ions [183]. The magnetic frame composite of MOFs adsorbents exhibited high reducibility when subjected to external magnetic pull [184]. In a study by Abdel-Magied and his team [185], a couple of magnetic metal–organic frameworks (Fe_3_O_4_@UiO-66-NH_2_ and Fe_3_O_4_@ZIF-8) were prepared and utilized for the removal of Pb and Cd. Fe_3_O_4_@UiO-66-NH_2_ showed removal efficiencies equal to 370 mg/g for Cd and 666.7 mg/g for lead, while Fe_3_O_4_@ZIF-8 exhibited removal efficiencies of the order of 714.3 mg/g for cadmium and 833.3 mg/g for lead. The adsorption procedure was found to be instinctive, endothermic and monolayer in nature, and both adsorbents preserved remarkable adsorption efficiencies after four cycles of usage. Another study by Abdelmoaty and coworkers [177] documented that modifying UiO-66 with melamine increased the adsorption of Pb (177.5 mg/g) and Cd (146.6 mg/g). The adsorption of Pb and Cd was physisorption-driven, and the procedure was spontaneous and endothermic at pH equal to 6. Metal–organic frameworks with ligands containing N atoms, such as CAU-7-TATB, exhibited improved adsorption capacity and high selectivity towards Pb^2+^ ions [186]. Furthermore, the presence of sulfur-functionalized MOFs enhanced the adsorption of Pb^2+^ ions from solutions. In a research using HS-mSi@MOF-5 and thiol-modified MOF-5 as adsorbents in a silica gel medium, HS-mSi@MOF-5 demonstrated a higher adsorption capacity with an *n* value > 1, indicating improved adsorption efficiency [187]. UiO-66-NH_2_-PAM-PET, which refers to MOFs modified with (C_3_H_5_NO)_n_ and (C_10_H_8_O_4_)_n_, effectively removed Pb^2+^ ions from water through ion exchange and electrostatic surface complexation. The adsorption capacity of melamine-modified MOFs was higher than pristine MOFs, with the acquired experimental results fitting the pseudo-second-order kinetic model. The interaction between amino groups and Pb^2+^ ions played a fundamental role in the adsorption procedure. Zheng and co-researchers [188] explored the removal of Pb^2+^ and Cr^4+^ ions from wastewater utilizing reusable metal–organic frameworks. The supreme adsorption efficiencies were 537.634 mg/g for Pb^2+^ (pH value equal to 7) and 787 mg/g for Cr^4+^ (pH value equal to 6). Adsorption equilibrium was achieved in 60 min for Pb^2+^ and 120 min for Cr^4+^, and regeneration was successful utilizing a CH_3_CH_2_OH-CH_3_COOH extractant subsequent to three cycles. The alignment of -OH groups and metal ions followed the hard–soft acid–base algorithm, substantiated by the interaction between imidazole N atoms and heavy metal ions, facilitated by the exposed active sites of the adsorbent.

Additionally, researchers have conducted studies to evaluate the adsorption efficiency of metal–organic frameworks for the removal of various heavy metals. More specifically, one study focused on synthesizing a modified magnetic MOFs for the removal of lead, mercury, and cadmium ions [189]. The synthetic route involved immobilizing tripeptide glutathione onto the magnetic MOF. Computational modeling was utilized to obtain the supreme adsorbent’s structure prior and after adsorption. The maximum adsorption efficiencies had been calculated equal to 397 mg/g for Pb^2+^, 393 mg/g for Cd^2+^, and 431 mg/g for Hg^2+^ ions. The modification of the adsorbent facilitated the removal of >90% of the heavy metal ions within 20 min, with the Langmuir isotherm and pseudo-second-order kinetics models providing a good fit. The excellent performance of the adsorbent could be attributed to the microcrystalline structure of the Zr MOF, which offers a large specific surface area for glutathione deposition.

Hg removal through adsorption utilizing metal–organic frameworks has been extensively studied, yielding various levels of fulfillment. For example, sulfide-modified metal–organic frameworks have demonstrated effectiveness in the adsorption and removal of Hg^2+^ because of strong bond development and affinity [190]. Zhou and co-researchers [191] studied the removal of Hg^2+^ from solution using ZIF-67, which exhibited a high adsorption efficiency equal to 1740 mg/g. The superior competence of this Co-based metal–organic frameworks can be ascribed to the robust coordination between Hg^2+^ ions and the N atoms of the imidazole ring in the ligand. In another research, mercaptoacetic acid groups were introduced to enhance Hg^2+^ removal using SH-MIL-68(In), while NH_2_-MIL-68(In) with NH_2_ groups provided more accessible sites for functionalization on the MOFs’ surface [192]. The inclusion of sulfur, acrylamide, carboxyl, and hydroxyl groups in metal–organic frameworks enhance the adsorption capacity for Hg^2+^, as Hg^2+^ exhibits strong binding affinity towards these groups. Tang and his team [193] documented the selective removal of Hg^2+^ from solution using a new Zr-based MOFs, achieving a concentration reduction < 0.88 ppb and an adsorption efficiency equal to 962 mg/g. The functionalization of metal–organic frameworks with C_6_H_4_S_4_ overcame the drawbacks of commonly used thio- and thiophene-functionalized metal–organic frameworks. The Zr-based metal–organic frameworks proved to be recyclable and reusable. A multifunctional adsorbent (UiO-66-EDTA) with amine and carboxyl groups in an octahedral structure achieved 99% removal of Hg^2+^ in competitive adsorption with Pb^2+^ and Eu^3+^ in an aqueous media [194]. The efficient removal of mercury ions can be attributed to the coordination between the metal ion, tertiary amines, and carboxyl groups. Additionally, a metal–organic framework synthesized using a C_15_H_12_N_4_ ligand and Co(SCN)_2_ (FJI-H30) demonstrated effective removal of mercury ions from a solution [195]. The mechanism of adsorption indicated that the adsorbed mercury ions bind to the S atom in the SCN group, leading to structural alterations in the mentioned metal–organic framework’s matrix. These structural modifications enhanced the bond between the described MOF and mercury ions, resulting in enhanced adsorption efficiency. Porphyrinic Zr metal–organic frameworks (PCN-221) fabricated through a solvothermal approach effectively removed Hg^2+^ from an aqueous medium, presenting adsorption efficiency equal to 233 mg/g in 30 min [196]. The occurrence of the porphyrin ligand in the PCN-221 matrix played a crucial role in the prosperous adsorption of mercury ions. The adsorption results fitted well with the pseudo-second-order kinetic equation, while the adsorption isotherms of mercury ions matched the Langmuir isotherm model.

Copper ions are present in two oxidation states (Cu^+^ and Cu^2+^), and the alterations between these states can produce -OH radicals and O^2−^, promoting their toxicity. While Cu can be valuable, excessive reception and exposure can cause damage to human organs [197]. The literature extensively covers the use of MOFs for copper adsorption. For instance, Subramaniyam and coworkers [178] employed an ecofriendly Zr-based MOF for the removal of copper ions from real water samples. The optimal adsorption efficiency was found to be 150 mg/g, resulting in a removal capacity equal to 50%. Yuan and his team [198] utilized water-stable MOF-199 to effectively eliminate copper ions from water, demonstrating a remarkably enhanced adsorption ability (7831.3 mg/g), surpassing the common values documented for other adsorbents used in copper adsorption. The increased adsorptive efficiency was ascribed to the adsorbent’s porous structure, as well as the coordination between Cu^2+^ ions and N atoms of the imine ligand. The mechanism of adsorption followed a monolayer type and the equilibrium results aligned well with the Langmuir isotherm. In order to increase copper ion removal from aqueous solutions, ZIF-8 was altered with three amino amines and graphene oxide [199]. This modification aimed to prevent MOF coagulation and enhance its adsorption capacity. ZIF-67 produced through physical mixture and lyophilization with bacterial cellulose/chitosan composite aerogel alteration (ZIF-67/BC/CH aerogel), effectively removed Cu^2+^ and Cr^6+^ ions from aqueous solutions [200]. The adsorption efficiencies for the adsorbent ranked 200.6 mg/g (Cu^2+^) and 152.1 mg/g (Cr^6+^), with improved stability of chitosan achieved by using bacterial cellulose fibers instead of chemical crosslinking agents. The adsorbent was successfully regenerated and reused with removal efficiencies equal to 72% (Cr^6+^) and 81% (Cu^2+^) after five subsequent adsorption cycles. Additional studies have documented the enhanced efficiency of various metal–organic frameworks in removing various heavy metals. 

Wang et al. [201] developed a novel macroporous membrane based on MOFs towards the efficient uranium extraction from seawater via constant filtration. The modification of UiO-66 with poly(amidoxime) (PAO) facilitated the good dispersion in a graphene oxide cotton fiber N,N-dimethylformamide solution. In comparison to nonmodified MOFs, the as-synthesized one was homogenously dispersed within the membrane due to its robust nature in the solution, resulting in a significantly enhanced uranium adsorption efficiency. Additionally, the membrane offered a facile and ongoing uranium adsorption procedure, unlike powdered MOFs. Thus, the uranium extraction efficiency of the proposed membrane achieved 579 mg/g in a 32 ppm U-added simulated seawater within just 24 h. Remarkably, the UiO-66@PAO membrane successfully removed 80.6% of UO^2+^_2_ from 5 L of seawater after 50 consequent filtering cycles, laying the foundation for an adaptable approach towards the design and synthesis of novel MOF-based adsorbents for effective U removal from seawater.

Summarizing, metal–organic frameworks and their composites exhibit favorable adsorptive features for heavy metal removal. Functionalization of such metal–organic frameworks can increase their environmental acceptability, strengthen their bonds with heavy metals, decrease coagulation and enhance the adsorption efficiency, resulting in increased removal of heavy metals from wastewater and aqueous solutions. Moreover, most adsorption kinetics can be illustrated by the pseudo-second-order model and the adsorption equilibrium is well described by the Langmuir isotherm model. Comparative results of heavy metal removal utilizing metal–organic frameworks and other adsorbents are summarized in Table 10, clearly indicating the relatively higher removal capacities of MOFs.

### 5.2. Adsorptive Removal of Fluoride

Fluoride pollution pertains to environmental contamination arising from an excess of fluoride and its compounds, either due to human activities or natural occurrences, such as industrial discharges involving fluorinated materials, the erosion of mineral-enhanced soil, volcanic activity, as well as the release of fluoride (F^−^) from marine aerosols [211,212]. This pollution poses a significant threat to both the environment and humans. Fluoride is a vital trace element essential for human health and constitutes a major component of bones and teeth, making it irreplaceable. Nevertheless, both excessive and insufficient ingestion of fluoride can lead to detrimental effects on human wellbeing [213]. Prolonged exposure to high levels of F^−^ through drinking water, air, or food can result in various health issues, including fluorosis, dental fluorosis, neurological disorders, Alzheimer’s disease, as well as suppression of enzyme malfunction within the body, impacting normal metabolism [214,215,216]. As a result, it is crucial to control the F^−^ content in drinking water to comply with the World Health Organization threshold of 1.5 mg/L [217].

Song and his team [217], assessed the comprehensive performance of a Ce(III)-4,4′,4″-((1,3,5-triazine-2,4,6-triyl) tris(azanediyl)) tribenzoic acid–organic framework (Ce-H3TATAB MOFs) towards the removal of excessive fluoride from aqueous solutions, while defluoridation was subsequently thoroughly investigated. The optimal adsorption efficiency was achieved when maintaining a 1:1 metal-to-organic ligand molar ratio. Additionally, the impact of pH and coexisting ions on the defluoridation efficiency was explored. Results indicated that Ce-H3TATAB MOFs exhibited mesoporous attributes with excellent crystallinity, and the quasi-second kinetic and Langmuir models were found to accurately describe the adsorption kinetics and thermodynamics, respectively. Notably, the maximum Langmuir adsorption efficiency was recorded equal to 129.7 mg/g at 318 K and pH value = 4. The team proposed that the adsorption mechanism primarily entailed ligand exchange, electrostatic interaction, as well as surface complexation. Furthermore, the most efficient fluoride elimination occurred at pH = 4, while under pH = 10, an elimination capacity equal to 76.57% was achieved, showcasing the broad applicability of the adsorbent. Finally, ionic interference studies demonstrated that the presence of PO_4_^3−^ and H_2_PO_4_^−^ within the H_2_O hindered defluoridation, whereas SO_4_^2−^, Cl^−^, CO_3_^2−^, and NO_3_^−^ facilitated fluoride adsorption, due to the ionic effect.

Ce-La MOFs composites were synthesized by Hu et al. [218] using the hydrothermal approach and were investigated as potential adsorbents towards the elimination of fluoride from aqueous solutions. They examined the optimal synthesis molar ratio, adsorption time, as well as the starting fluoride concentration. Moreover, the influence of coexisting ions on the elimination effectiveness and regeneration of the adsorbents was also explored. The research’s findings indicated that the most effective conditions for defluoridation, utilizing the proposed MOF composite material, were achieved at a pH value equal to ≈3; with a molar ratio of La(NO_3_)_3_, Ce(NO_3_)_3_, and C_8_H_7_NO_4_ at 3:1:2; a starting F^−^ concentration equal to 10 mg/L; and an adsorption period of 2 h. Under these specific conditions, the elimination capacity was >90%. The interference experiments demonstrated that the adsorbent exhibited enhanced selectivity for F^−^ with the majority of added ions, having only a negligible influence on the adsorption efficiency, while a few of them displayed an inhibitory effect. Kinetic and thermodynamic studies indicated that the adsorption process followed the Langmuir and pseudo-second-order models, with a maximum Langmuir adsorption efficiency equal to 138.64 mg/g (50 °C). The observed adsorption mechanism included a combination of ion exchange and electrostatic interaction mechanisms. Regarding the regeneration cycle studies, the aforementioned composites indicated favorable regeneration efficiency. More specifically, even after four regeneration cycles, the adsorption efficiency was equal to 36.25 mg/g, achieving 73% elimination capability (C_0_ = 10 mg/L).

In a study by Yang and his team [219], La-altered ZIF-8 hybrid materials (La-ZIF-8) with varying La contents were successfully synthesized using a solvothermal approach and were subsequently utilized towards fluoride recovery from wastewater. The defluoridation capability of the proposed hybrid material under several conditions was evaluated through static batch experiments. The optimized hybrid material (La/Zn molar ratio equal to 4:1) demonstrated the most effective defluoridation ability. The fluoride adsorption was significantly improved from 1.85 mg/g for pure ZIF-8 to 49.5 mg/g for the La-ZIF-8 at C_0_ = 10 mg/L. Furthermore, the acquired data indicated that the maximum fluoride adsorption for the aforementioned hybrid material was 224.72–230.95 mg/g within the temperature range of 30–50 °C, pH = 4, and C_0_ = 80 mg/L. The adsorption of F^−^ on the hybrid material followed the Langmuir isotherm (R^2^ > 0.99), as well as the pseudo-second-order kinetic model (R^2^ > 0.999). The presence of various cations and anions had only a negligible impact on F^−^ elimination efficiency, highlighting the high selectivity of the mentioned hybrid material towards fluoride. Overall, the resulting experimental data indicated that the La-ZIF-8 hybrid material shows promise as a potential adsorbent for addressing fluoride pollution.

### 5.3. Adsorptive Removal of Organic Dyes

Dyes induce changes in color on a surface by undergoing processes that can modify the composition of the colored substance. Two types of dyes exist: ionic and nonionic. Ionic dyes can be categorized as cationic or anionic based on the presence of positively or negatively charged ions, respectively, when in a H_2_O solution. Examples of cationic dyes feature acid, reactive, and direct dyes, and anionic dyes encompass malachite green, methylene blue, methyl orange, and rhodamine B [220]. Textile industries generate significant wastewater amounts during manufacturing, which is often characterized by a high concentration of dyes. Removing dyes from wastewater using conventional methods is challenging due to their high solubility in water [221]. Dyes, as contaminants in water, pose considerable health risks such as allergies, blindness, and chemosis, as well as ecological concerns including enhanced COD, limited DO, and inhibition of microbial evolution [141].

Several researches have explored the utilization of metal–organic frameworks for the elimination of various dyes [222,223,224,225,226,227,228,229,230,231,232]. This paragraph focuses on the adsorptive elimination of selected dyes from solution to showcase the adsorption efficiency, reusability, as well as kinetic mechanisms of metal–organic frameworks. In research by Mohd and his team [226], malachite green was eliminated from a solution through a coprecipitation method to synthesize a poly-o-anisidine/zinc oxide metal–organic framework (PAZ@ZIF-8). PAZ@ZIF-8 exhibited malachite green removal capacity equal to ≈96%, surpassing ZIF-8 and POA/ZnO, that achieved removal capacities equal to 34% and 61%, respectively. The results of the adsorption studies for PAZ@ZIF-8 matched the Langmuir isotherm model and the sorption kinetics adhered to the pseudo-second-order model. The optimal adsorption efficiency was determined to be 613 mg/g. PAZ@ZIF-8 demonstrated good reusability as it maintained a dye removal rate of around 90% after three cycles. Another study conducted by Mahreni and his team [233] explored the ability of a composite material consisting of calcium alginate and MOFs for removing malachite green from a solution. The results indicated an 84.5% removal efficiency when the contact time was set at 120 min, the dye was characterized by an initial concentration equal to 5.5 mg/L, while the adsorbent dosage was 0.06 g/L. The adsorption procedure aligned with the alternated pseudo-first-order kinetic model.

Mohammadi and colleagues [234] conducted a comparative analysis between nickel-based and iron-based MOFs to evaluate their effectiveness in sequestering malachite green and alizarin red from solution. Their findings revealed that the Fe-based metal–organic framework outperformed the Ni-based metal–organic framework, exhibiting removal capacities of 88% and 6% for malachite green and alizarin red, respectively, in contrast to the efficiencies of 9% and 5% documented for the Ni-based metal–organic framework. Wu and co-researchers [235] fabricated two novel Zn-based metal–organic frameworks through the solvothermal approach, both of which demonstrated exceptional capacity for selectively adsorbing and photocatalytically degrading malachite green. The adsorption process was accurately illustrated by the Langmuir isotherm, as well as the pseudo-second-order kinetic model. Congo red dye has garnered significant attention and Sohrabnezhad and his team [232] utilized a facile and rapid procedure to develop sphere-shaped MOF-74 that was subsequently employed towards the photodegradation of Congo red. Maximum removal capacity equal to 94% was achieved after 60 min, adsorbent dosage equal to 1 g/L, and dye concentration equal to 10 mg/L. The exceptional removal capacity of the mentioned metal–organic framework was ascribed to its substantial specific surface area, as well as its chemical and thermal stability. Koppula and coworkers [231] employed the solvothermal approach to develop MOF-2@Al_2_O_3_ and evaluated its effectiveness in a fixed bed adsorption column for the removal of Congo red, methyl orange, as well as methylene blue. By optimizing various process parameters (contact time, flow rate, bed height, pH, initial concentration), they achieved removal capacities of ≈100% for all studied dyes. Moreover, Gao and his team [230] synthesized a 3D Zn-based metal–organic framework utilizing the solvothermal approach, which exhibited exceptional adsorption efficiency towards elimination of Congo red and methyl orange from solution. The adsorption of Congo red was enabled by the gravitational settling of enlarged molecules due to the hydrogen bonding between the amine group in Congo red and the -OH group within the structure of the metal–organic framework.

Another study focused on removing azo dyes from a solution using Ni(II)-doped MIL-101(Cr) [236]. The adsorption efficiency of this materials for Congo red and methyl orange was greater than that of pristine MIL-101, with increases of 31.4% and 23.4%, respectively. However, the adsorption capacity towards acid chrome blue K decreased by 50.2% after the doping. The introduction of Ni(II) resulted in structural and morphological defects in the MOFs, enhancing electrostatic interactions and adsorption efficiency towards Congo red and methyl orange. The reusability potential of Ni(II)-doped MIL-101(Cr) presented 10% reduction in efficiency after four cycles. In another study, a protonated Zn MOF was used to remove several anionic dyes from a solution [91]. The described metal–organic framework exhibited increased adsorption efficiency towards the examined dyes, ranging from 522.83 to 2402.82 mg/g. The adsorption procedure pursued pseudo-second-order kinetics and conformed to the Langmuir and Sips models. The interaction between the protonated Zn MOF adsorbent and the anionic dye can be most accurately characterized as electrostatic attraction with surface adsorption.

Methylene blue was effectively eliminated from water by utilizing a Ni-based 2D nanosheet metal–organic framework, which showed comparable adsorption capacity (765 mg/g) to highly porous Ni-MOF-199 (798.0 mg/g) [237]. The layered nanostructure of the Ni MOF facilitated dye species’ diffusion. The photodegradation of rhodamine B upon UV light irradiation was investigated utilizing Ni-, Fe-, and Ti-based metal–organic frameworks by Pattappan and co-researchers [238]. The Fe MOF exhibited enhanced photodegradation effectiveness (90%) in comparison to the Ti MOF (50%) and Ni MOF (9%) under pH value equal to 7 and contact time equal to 90 min. The adsorption procedure utilizing the Fe-based metal–organic framework conformed to a first-order-kinetics, while no considerable decrease in the removal capacity was documented after four cycles. Pham et al. [239] synthesized a Bi MOF for the elimination of rhodamine B from a solution, achieving a removal efficiency of 98% at optimized adsorbent dosage, dye concentration, and irradiation time. The tested adsorbent indicated a negligible decrease (5%) in its removal capacity after four subsequent adsorption cycles.

Lately, great interest has been directed towards the implementation of advanced oxidation processes (AOPs) to degrade organic pollutants instead of relying solely on adsorption methods [50,161]. AOPs utilize extremely reactive radical species, like •OH, H_2_O_2_ and •SO_4_^−^, to oxidize organic pollutants into less noxious substances. Notably, there has been a particular focus on the degradation of dyes using visible-light irradiation. Namely, Feng and his team [240] investigated the efficacy of MIL-53(Fe) as a photocatalyst for degrading methylene blue under visible light conditions. A degradation rate equal to 87% was observed for MB, in comparison to only 7% without the presence of the photocatalyst. Similarly, Araya and his team [241] modified MIL-53(Fe) using anionic and cationic resins in order to develop functionalized metal–organic framework photocatalysts. They evaluated the ability of these photocatalysts in degrading rhodamine B upon visible light irradiation. Upon 120 min of irradiation, the degradation capacity of cationic resin-modified MIL-53(Fe) reached 96%, while virgin MIL-53(Fe) achieved only 24% degradation. Additionally, the cationic resin-modified MIL-53(Fe) exhibited selectivity towards degrading anionic dyes, while the anionic resin-modified MIL-53(Fe) indicated selectivity towards cationic dyes.

ZIF-67@wood composite materials were synthesized [242] through the in situ growth of ZIF-67 on wood, which were subsequently carbonized to create magnetic WC-Co composites to be utilized towards the adsorption of Congo red and methylene blue dyes. The unique hierarchical porous structure of wood enabled efficient transfer of dye solutions, facilitating full contact between magnetic core–shell nanoparticles and dyes. The adsorption efficiency of the synthesized composite material was 1117.03 mg/g and 805.08 mg/g for Congo red and methylene blue, respectively. When the dye concentration reached 1200 mg/L, the proposed composite achieved a remarkable elimination efficiency equal to 99.98% under gravity. Importantly, the composite material demonstrated enhanced reusability, while the adsorbed dyes could be easily eliminated through burning. The aforementioned cost-effective and recyclable material, boasting ultrahigh adsorption efficiency, could potentially replace commercially available activated carbon and find widespread application in practical wastewater treatment.

The utilization of •SO_4_^−^ radical-based AOPs combined with various metal–organic frameworks has gained interest lately [49]. A composite (Fe_3_O_4_@MIL-101) photocatalyst was synthesized by Yue et al. [243] towards the triggering of persulfate in the degradation of Acid Orange 7. They observed that MOFs facilitated the production of •SO_4_^−^ and •OH radicals, with •SO_4_^−^ radicals being the primary oxidizing species accountable for Acid Orange 7 degradation. Furthermore, alternative advanced oxidation processes, like Fenton and photo-Fenton, have been catalyzed via metal–organic frameworks and MOF-based materials [244]. 

According to the research reviewed in this paragraph, it is evident that metal–organic frameworks serve as efficient adsorptive material towards the removal of dyes from H_2_O solutions. Several composites have been added into the MOFs’ matrixes in order to achieve specific objectives, such as increased adsorption of dyes from H_2_O solutions or wastewater. The majority of researches conform to the Langmuir adsorption isotherm in explaining the equilibrium results. However, the pseudo-second-order kinetic model is frequently employed as well. Table 11 compiles the analogous findings from chosen studies on dye removal employing MOFs and other examined adsorbents, as documented in the literature.

### 5.4. Adsorptive Removal of Antibiotics

Antibiotics play a crucial role in promoting good health by being widely used to treat microbial infections. It is estimated that approximately 5–90% of active antibiotic compounds consumed are eliminated from the body through feces and urine [252]. However, these antibiotics can enter the environment and water bodies following excretion, leading to environmental pollution and raising concerns. The pollution caused by antibiotics is an escalating worldwide ecological concern given the adverse impacts associated with overexposure to these drugs, as well as drug resistance [253]. Consequently, there is an urgent requirement for effective antibiotic elimination methods to safeguard the environment and preserve ecological equilibrium.

Various methods are commonly employed towards the elimination of antibiotics, including membrane filtration, biological treatment, adsorption [254], electrochemical treatment [255], advanced oxidation technology [256], as well as photodegradation [257]. Among these, adsorption and photodegradation are particularly favored due to their notable advantages such as high efficacy, straightforward synthesis, low cost, sustainability, as well as versatility in application [258]. However, these methods do have certain limitations that hinder their efficiency and widespread implementation. These limitations encompass factors such as removal effectiveness, functional intricacy, and the formation of potentially carcinogenic and mutagenic byproducts. When it comes to photocatalytic degradation, the utilization of irradiation is constrained by the requirement for ultraviolet light to activate photocatalysts with wide band gaps. Additional limitations entail decreased quantum effectiveness and an increased propensity for electron–hole pair recombination in catalysts. Furthermore, concerns arise from the restricted specific surface area and pore volume of adsorbents and catalysts, which reduce the quantity of active sites and their availability [253]. Consequently, there is an imperative requirement for the advancement of more effective and resilient methods in order to address the removal of antibiotics from the environment.

MOFs have garnered significant attention as favorable materials towards the effective elimination of antibiotics from water thanks to their unique characteristics. These include their large specific surface area, homogeneous porosity, tunability of pore volume and porosity, and structural robustness, making them subject of extensive research on antibiotic adsorption from aqueous solutions [258]. In research conducted by Zhao and coworkers [259], focus was placed on the elimination of chloramphenicol antibiotic from wastewater via a novel MOF-based adsorbent called PCN-222. Chloramphenicol can have adverse consequences like fatal bone marrow depression and aplastic anemia when present in drinking water [260]. The synthesis of PCN-222 was carried out on a large scale under normal pressure through refluxing. Subsequently, its performance was assessed for chloramphenicol removal from aqueous solutions. The research revealed that the aforementioned material exhibited superior adsorption efficiency compared to other MOFs and non-MOF adsorbents. This MOF-based material displayed increased adsorption efficiency and achieved rapid adsorption equilibrium. At low concentrations, PCN-222 could eliminate approximately 99% of chloramphenicol from H_2_O. The exceptional elimination capacity of PCN-222 was ascribed to its exceptional pore structure and H-bonding and electrostatic interactions. According to the research’s results, such materials could be employed for effective elimination of chloramphenicol and other antibiotics from aquatic environments. 

Zhou and coworkers [261] assessed the efficiency of a Fe MOF for the elimination of tetracycline and norfloxacin, both in single and competitive adsorption systems. The adsorption efficiencies of the Fe MOF for tetracycline and norfloxacin were found to be decreased in the competitive adsorption system in relation with the single one. The described material exhibited stability and reusability. 

Moreover, Xia and his team [262] investigated the adsorptive elimination of tetracycline from H_2_O solutions utilizing various types of Zr-based MOFs, including UiO-66, NU-1000, and MOF-525. The adsorption kinetics fitted to the pseudo-second-order model and the Sips model presented the optimal fit for the adsorption isotherm. Among the studied MOFs, NU-1000 exhibited the most enhanced adsorption rate, while MOF-525 demonstrated the most increased adsorption efficiency. More specifically, the Sips adsorption efficiencies for tetracycline using Zr MOFs had been reported as 807 mg/g for MOF-525, 356 mg/g for NU-1000, and 145 mg/g for UiO-66. In general, the obtained values exceeded those of commonly used adsorbents, indicating the potential of Zr MOFs in water treatment for tetracycline removal. 

Chen and his team [263] studied the elimination of ciprofloxacin, achieving a 90% elimination capacity within 30 min. Additionally, the adsorbent demonstrated reusability, maintaining an 85% elimination effectiveness upon four cycles. Furthermore, Kim and co-researchers [264] successfully adsorbed tetracycline and ciprofloxacin utilizing an Al-based MOF-rGO-immobilized alginate adsorbent in a batch system. At temperature equal to 40 °C, pH value equal to 7 and contact time 12 h, the adsorbent exhibited the maximum adsorption efficiencies of the order of 43.76 mg/g for tetracycline and 40.76 mg/g for ciprofloxacin, respectively. Chaturvedi and co-researchers [265] synthesized Fe-based MOFs (MIL-100(Fe)), utilizing a solid approach for the elimination of levofloxacin from water. The acquired Langmuir isotherm data indicated increased adsorption efficiency (87.34 mg/g), with electrostatic interaction and hydrogen bonding being the predominant mechanisms during the adsorption procedure.

In addition, Zhao and colleagues [266] produced a Zr-based MOF called PCN-777 as an adsorbent towards the removal of cephalexin from wastewater. The mentioned MOF exhibited high specific surface area (2004 m^2^/g), enhanced porosity, increased pore size, as well as exceptional water stability. PCN-777 showed maximum adsorption efficiency equal to 442.48 mg/g. The adsorption procedure pursued the pseudo-second-order as well as Langmuir isotherm models. The researchers also considered the reusability of the MOF, which could be achieved through a simple method. These findings indicate the suitability and potential application of PCN-777 as an efficient adsorbent for antibiotic elimination from wastewater, as well as its potential for guiding the design and synthesis of novel adsorbents.

Sulfamethoxazole constitutes a widely utilized sulfonamide antibiotic, which threatens human health and the environment due to its low biodegradability. Cheng and colleagues [267] developed a surface molecular imprinted polymer on a MOF (MIP-IL@UIO-66) towards selective and fast adsorption of sulfamethoxazole from H_2_O solutions. The aforementioned MOF exhibited a maximum adsorption efficiency equal to 284.66 mg/g, achieving adsorption equilibrium within 10 min. The adsorbent demonstrated excellent reusability and stability, with adsorption efficiency > 92% even after five reusability cycles. The study highlighted the good adsorption efficiency, selectivity, rapid adsorption rate, and reusability of the synthesized MOF, suggesting its potential for enhancing the removal of sulfamethoxazole and other antibiotics from water.

AOPs have been the subject of investigation for eliminating antibiotics from wastewater [121]. Namely, Mao and his team [268] developed a highly effective magnetic carbon derived from Co-based MOF (Co@C-600) and assessed its capability to activate peroxymonosulfate towards the oxidative degradation of oxytetracycline (OTC). The results indicated that over 89% of OTC had been degraded within 15 min. The degradation procedure involved a combination of radical (•OH and •SO_4_^−^) and nonradical mechanisms, as evidenced by electron paramagnetic resonance tests. In a similar study, Yang and co-researchers [269] fabricated a porous magnetic derivative of MIL-101 (Fe-Co) and employed it for activating peroxymonosulfate to degrade chloramphenicol in solution. Thorough degradation of the antibiotic had been achieved within 120 min utilizing catalyst concentration equal to 0.1 g/L (pH = 8.2). Hydroxyl and sulfate radicals were identified as the primary oxidative species accountable for chloramphenicol’s degradation. Additionally, ZIF-8 derivatives were prepared to activate peroxymonosulfate towards degradation of levofloxacin upon visible light irradiation [270]. Within 1 h upon irradiation, the degradation capacity of levofloxacin reached 87.3%. The mechanism analysis revealed the involvement of holes, •SO_4_^−^, and •O_2_^−^ radicals in facilitating the degradation process.

A recent literature review indicated that photocatalytic materials like TiO_2_, CNTs, and GO have been utilized for antibiotic removal; however, their performance is hindered by limited light usage and decreased specific surface area. Moreover, antibiotics’ recalcitrant nature exacerbates the challenge. In contrast, the researches reviewed within this paragraph demonstrate the superior efficiency of MOF-based photocatalysts due to their narrower band gap, reduced electron–hole recombination ratio, and enhanced light use effectiveness [271]. Consequently, metal–organic frameworks and their derivatives have emerged as favorable materials for photocatalytic antibiotic degradation. Nevertheless, certain MOFs exhibit low adsorption capacity, prompting efforts to enhance their properties. Notable improvement strategies include incorporating functional groups like NO_2_, NH_2_, NO_3_, SO_3_H, Br, and Cl into the MOFs’ structural matrix, as well as metal doping, polymer coupling and hierarchical pore development [272].

Table 12 presents selected research on antibiotic elimination employing metal–organic frameworks and other adsorbents. Although promising impacts have been achieved utilizing several MOF-based materials for antibiotic removal, further research is required to fabricate exceptionally effective approaches with enhanced antibiotic elimination capabilities and improved degradation performance.

### 5.5. Adsorptive Removal of Pesticides and Herbicides

Pesticides play a crucial role in promoting sustainable agriculture by reducing crop losses and improving yields. They solved problems relating to diseases and pests, but they also introduced environmental problems. When organophosphorus pesticides are washed off from agricultural fields, they can contaminate surface and groundwater as well as agricultural produce, posing health risks when ingested [279]. Organophosphorus pesticides are highly toxic to humans and are regulated in many countries. It is critical to fiend efficient ways to detect and remove these pollutants. Different conventional techniques have been utilized to eradicate these contaminants, which are mentioned above [80,280,281]. Adsorption has shown great promise due to its simplicity, cost-effectiveness, and efficiency. As a result, there have been numerous studies investigating novel adsorbents like metal–organic frameworks (MOFs) for dealing with pesticides. 

Numerous studies have explored the utilization of pure metal–organic frameworks for effectively removing pesticides from water. The team of Mirsoleimani-Azizi [282] employed mesoporous MIL-101(Cr) in static adsorption system for continuous removal of diazinon. They observed a removal efficiency of 92.5% for concentrations of 150 mg/L. The large diazinon molecules where able to be absorbed within the mesoporous structure of MIL-101(Cr). Elsewhere, MOF based on copper was used to remove the carcinogenic and toxic pesticide 14C-ethion [283]. The highest adsorption achieved was 122 mg/g, with optimal conditions of 150 min contact time, 25 °C temperature, 0.425 g/L adsorbent dosage, pH equal to 7, as well as pollutant concentration equal to 75 mg/L. In addition, they compared the absorption capabilities of ZIF-67(Co) and ZIF-8(Zn) for the pollutants ethion and prothiofos [284]. In the case of ZIF-67(Co), the absorptions were measured at 211 mg/g and 261 mg/g, respectively, while ZIF-8(Zn) exhibited adsorption of 279 mg/g for ethion and 367 mg/g for prothiofos. The dissimilar adsorption capacities were ascribed to the distinct coordination levels among pesticides and metal ions within the MOFs. Recently, Yang Li and his team [285], developed a novel material for the elimination of organophosphorus pollutants in a solid phase. The synthesis involved surface molecular imprinting, utilizing fenthion for a template, methacrylic acid as a functional monomer, and for a crosslinking agent ethylene glycol dimethacrylate was used. The resulting framework had a core–shell structure with a shell that exhibited specific recognition and fast adsorption capacity for organophosphorus pesticides. These studies proved that is possible to design highly efficient adsorbents for various pollutants. In different studies by Ashouri and team [286], an activated extruded UiO-66-based metal–organic framework (AE-MOF-UiO-66) was synthesized towards the adsorption of diazinon from water. They managed to increase the performance of the regular MOF UiO-66. In addition to the impressive adsorption capabilities of the material, it is possible to separate and reuse it. 

Sheikhi et al. [287] conducted a separate study where they explored the elimination of methyl parathion and diazinon organophosphate pesticides using an MOF called BSA/PCN-222(Fe). Bovine serum albumin was used for the functionalization of the framework, through a straightforward encapsulation process. The composite exhibited remarkable adsorption capabilities, removing 96% of the pollutants. At a neutral pH, 370.4 mg/g of methyl parathion and 400 mg/g of diazinon where absorbed after 180 s. The improved adsorption capability of the hybrid material can be ascribed to the existence of numerous binding sites within it. This study indicates that the hybrid material is highly efficient and recyclable, making it suitable for removing organophosphate insecticides from aqueous solutions.

Research conducted by Lu et al. [288] focused on eliminating azole fungicides from H_2_O through the utilization of a magnetic metal–organic framework functionalized with lignosulfonate. They synthesized a new magnetic composite called Fe_3_O_4_@LS@ZIF-8 by integrating lignosulfonate (LS) and ZIF-8 onto the Fe_3_O_4_. The composite exhibited superior adsorption, achieving removal efficiencies of up to 99%. The absorption was best explained by the Langmuir isotherm and pseudo-second-order models, indicating that the main mechanism was chemical adsorption. According to thermodynamic investigations, the adsorption of azole fungicides onto Fe_3_O_4_@LS@ZIF-8 was found to be endothermic and spontaneous. The mechanism of adsorption was attributed to hydrogen bonding, π–π interaction, and covalent bonding. The study further showcased the stability of Fe_3_O_4_@LS@ZIF-8 even after undergoing multiple cycles of adsorption and desorption. This finding highlights the cost-effectiveness and efficiency of the adsorbent for the elimination of azole fungicides from H_2_O.

Ma and coworkers [289] studied the magnetic solid-phase separation of triclosan (TCS) and triclocarban (TCC) fungicides from water, using a Zr-based magnetic MOF. For the synthesis, a solvothermal process was used. UiO-66 were immobilized onto Fe_3_O_4_@SiO_2_ particles. The magnetic metal–organic frameworks showcased exceptional thermal stability, favorable magnetic properties, consistent morphology, high capacity for adsorption, rapid attainment of equilibrium, and effortless magnetic separation when utilized for fungicide removal. The mechanism adhered to the pseudo-second-order kinetic model and Langmuir adsorption isotherm model. The theoretical calculations of the study were confirmed by the experiments. The MMOFs’ adsorption mechanism encompassed various interactions, including π–π interaction, hydrogen bonding, hydrophobic interaction, and coordination. Among these, hydrogen bonding was identified as the primary driving force for adsorption across different pH values. Additionally, the magnetic MOFs lost less than 20% of capacity after eleven cycles. The researchers concluded that MMOFs are suitable adsorbents for removing fungicides in wastewater treatment due to their excellent adsorption properties and reusability.

In summary, the literature analysis reveals that simple metal organic frameworks are effective in removing pesticides from water, displaying favorable kinetics and high adsorption capacities. Functionalized MOFs, including magnetic MOFs, offer enhanced adsorption properties and facilitate efficient removal of pesticides from solutions. Anionic or cationic agents can improve the abilities of functionalized MOFs for adsorbing corresponding pollutants. Furthermore, magnetic MOFs can easily be recovered and reused.

### 5.6. Adsorptive Removal of Endocrine-Disrupting Substances

Endocrine-disrupting compounds (EDCs) have become a significant environmental concern as they can cause hormonal imbalance. These compounds are of emerging concern due to their bioaccumulative and persistent nature. EDCs can disrupt the normal hormonal balance in the body, affecting fertility, reproductive health, and neurodevelopmental growth. Additionally, they can have adverse effects on many ecosystems [290]. EDCs are commonly found in various products including many chemicals, oils, and even home appliances and products [291]. They enter the human body through different pathways, including inhalation of air, consumption of contaminated water and food, and skin contact. In addition to anthropogenic sources, natural sources of EDCs include chemicals like estrogens, androgens, and phytoestrogens derived from plants. These compounds from plant origins can also exhibit endocrine-disrupting properties [292]. Given the potential health risks and environmental impacts associated with EDCs, it is crucial to raise awareness, monitor their presence, and develop effective strategies to mitigate their effects on human and environmental health.

The development of effective factionalized adsorbent materials is crucial for the mitigation of these pollutants. Metal–organic frameworks (MOFs) have gained attention as a better option than regular adsorbents for the purification of aqueous environments from EDCs. 

A lot of published research exists on the subject of adsorption of these pollutants utilizing metal–organic frameworks [237,293,294]. In one study, Samuel and his team [295] conducted experiments on the elimination of pharmaceutical products that are considered pollutants, like ibuprofen and acetaminophen. For their experiments, they factionalized metal–organic frameworks (MOF-199@NH_2_) with amines. The mechanism of adsorption was presented using the pseudo-second-order kinetic model and the Langmuir isotherm model. The results showed that 187.97 mg/g and 125.45 mg/g of ibuprofen and acetaminophen were absorbed, respectively. The results indicated that CuMOF@NH_2_ could be effective in removing dangerous contaminants from wastewater. Liu and his team [296] studied the removal of perfluorooctanoic acid (PFOA) using a type of MOF named MIL-101(Cr). The MOF had been modified with amine and quaternary amino groups both during the original assembly and after that. The PSM-synthesized MIL-101(Cr) exhibited an increased pollutant adsorption capacity of 1.82 mmol/g in comparison to 1.19 mmol/g for the PAM-synthesized MIL-101(Cr). The difference in the abilities of the synthesized materials was attributed to the presence of aromatic amines at the pore apertures, which hindered the entrance of target molecule into the porous structure. These studies demonstrate the potential of MOFs for removing EDCs from water system using their absorbance capabilities. Continuous research in this area can lead to the design of effective and efficient adsorbents for mitigating the impacts of EDCs on human and environmental health.

In a study by Park and Jhung [297], a metal–organic framework factionalized with amine (MIL-101-NH_2_) was used for the removal of bisphenol S from water. The MIL-101-NH_2_ adsorbent showed high effectiveness, achieving adsorption of 513 mg/g at a neutral pH. It also demonstrated good reusability and recyclability through simple ethanol washing. The adsorption mechanism is based on hydrogen bonding and π–π interactions, according to the researchers, indicating the potential of aminated MOFs as promising adsorbents for sulfonyl-containing organics found in aquatic systems.

In their study, Sini and coworkers [298] explored the adsorption capabilities of metal–organic framework sorbents factionalized with UiO-66 and fluorinated UiO-66 (F4) for removing perfluorinated molecules (PFOA and PFOS) from water environments. The materials were stable in water and achieved fast adsorption. The maximum adsorption for UiO-66 was 254 mg/g for PFOA and 160 mg/g for PFOS, whereas the fluorinated material was better, with a capacity of 467 mg/g for PFOA and 388 mg/g for PFOS. The proposed mechanism was based on van der Waals interactions between the fluorinated functionality of the MOFs and the fluorine present in the pollutants.

Chen et al. [299] studied the removal of used nitrophenol and bisphenol using an MOF-derived magnetic porous carbon-based sorbent. The adsorption achieved was 113 mg/g for nitrophenol and 49.43 mg/g for bisphenol A. Electrostatic and hydrogen bonding interactions seem to be responsible for this action. MIL-101(Cr) and MIL-100(Fe) metal–organic frameworks were also studied as adsorbents of bisphenol A from aqueous solution [300]. MIL-101(Cr) exhibited better adsorption uptake (252.5 mg/g) and adsorption rates compared to MIL-100(Fe), attributed to its larger pore volume and surface area.

One area that has been studied recently is the photodegradation of compounds that disrupt endocrines, using hybrid materials like metal–organic frameworks. Gong and his team [301] synthesized a hybrid material using graphitic carbon nitride (g-C_3_N_4_-MIL-101(Fe)) and found that it showed approximately nine times higher degradation efficiency for bisphenol A (BPA) compared to pristine graphitic carbon nitride. 

Liang and coworkers [302] used MOFs to develop a catalyst capable of inducing degradation of pharmaceutical pollutants like BPA, ibuprofen, and theophylline when irradiated with visible light. Two substances (ibuprofen/theophylline) where completely eliminated, while a little less than 40% of BPA remained. These studies highlight the potential of MOF-based adsorbents and photocatalysts for the efficient elimination and degradation of EDCs. Overall, pristine, functionalized, and composite MOFs have shown excellent performance in sequestering EDCs from aqueous solutions, exhibiting high adsorption capacities and short adsorption times. Functionalized and composite MOFs often offer enhanced efficiency and reusability, making them suitable for large-scale industrial applications in the removal of EDCs.

### 5.7. General Limitations 

Despite the positive results achieved through the utilization of several metal–organic framework-based substances in water decontamination, there are still limitations and challenges that need to be addressed. Improving the efficiency of approaches and sorbents to enhance elimination and degradation efficiencies remains a difficult task. Numerous strategies have been suggested to enhance the adsorption effectiveness of MOFs, such as incorporating metal dopants (like copper and nickel), integration with polymers (like MF and GO), introducing functional groups (like Cl, Br, and NH_2_) and modifying hierarchical pores using methods like polyelectrolyte-assisted structuring, thermal reflux, and HCl treatment [265]. 

Additionally, the increased cost of organic ligands used in MOF synthesis presents a significant challenge, making it less cost-effective and limiting its practical applications. One possible solution is to explore recycled organic ligands, such as C_8_H_6_O_4_ retrieved from waste bottles, as alternatives to commercial ligands [258]. 

Another limitation lies in the form and consistency of MOFs, as their powdered state often hampers transfer, separation, as well as recovery procedures, thereby reducing their adsorption effectiveness [286]. Overcoming this issue can be achieved through appropriate shaping and activation of the adsorbent materials, mitigating the challenges associated with powdered MOFs while maintaining their adsorption capacity [303]. Activation processes help enhance the porosity of such materials via removing guest molecules from the framework, simultaneously preserving its robustness [304].

There is an imperative necessity to synthesize MOF-based sorbents possessing favorable structural characteristics and enduring H_2_O stability [292]. It is essential to conduct comprehensive and meticulous research to assess as well as endorse the effectiveness of water-stable metal–organic frameworks and their derivatives. Various strategies are being explored to design water-stable MOFs, including the integration of water-resistant functional groups like trifluoromethoxy into linker scaffolds (e.g., IRMOF-1) [305], the fabrication of composites using carbon materials [306], as well as fluorination [307], among others. 

Additionally, extended study is needed to deepen our awareness of the reliability and robustness of metal–organic frameworks within more complex settings like acid/base solutions and the interactions among different linkers and metal ions to fabricate acid- and base-resilient metal–organic frameworks [292]. There is concern for MOF toxicity in H_2_O, as the leaching of adsorbents can potentially lead to contamination that surpasses the severity of the initial pollutants [289]. To address this, ecofriendly adsorbents and the recovery of adsorbents post-adsorption are advocated. This can be achieved by embedding magnetic materials within the adsorbents, enabling easy separation from the aqueous solution using an extraneous magnetic influence, eliminating the necessity of centrifugation and/or filtration. Furthermore, in order to moderate the potential of subsequent pollution caused by the leaching of noxious metals into the environment, it is recommended to use less hazardous metals like Zr, Mg, Ca, Fe, Ti, and Al as precursor metals for MOF fabrication [308]. MOF development should also employ environmental-friendly and low-cost biocompatible linkers, as well as green solvents like C_3_H_6_O, CH_3_CH_2_OH, H_2_O, and C_4_H_8_O_2_. In addition, it is crucial to conduct thorough lifecycle analyses and ecotoxicological assessments on metal–organic frameworks, particularly concerning their disposability [309].

## 6. Biomedical Applications of MOFs

**Figure 10:**
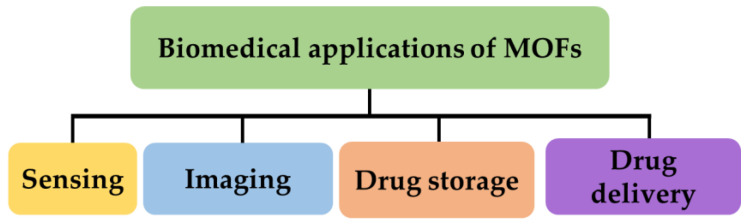
Biomedical applications of MOFs.

Among the aforementioned applications, MOFs are very promising in biomedical applications. Several studies have focused on their use in imaging, sensing, drug storage, and drug delivery platform development (Figure 10), since the physicochemical and structural properties of them allow biodegradability, functionality, and high loading potential [310]. However, there are still some issues regarding their biocompatibility, stability, and toxicity that remain to be further improved in order to be widely used in clinical routine [311]. 

In the following subsections, some characteristic applications of MOFs focusing on the diagnosis and treatment of cancer, diabetes, wound healing, and brain and neurological disorders are discussed by comparing their performance, highlighting the potential use of these materials in these fields of biomedicine, and mentioning some limitations and the need for their optimization. 

### 6.1. MOFs in Cancer Disease

Unfortunately, cancer is still a leading cause of death worldwide and breast, lung, colon, rectum, and prostate are among the most common neoplastic types. Early and accurate detection allows for an efficient therapeutic outcome [312]. Thus, the science community is consistently pursuing novel forms of therapy, timely identification, and prompt discovery to counteract such ailments [313]. Particularly, several studies focus on better understanding of the molecular mechanisms that mediate tumorigenesis [314], oxidative-stress [315], aging and cellular senescence [316], and numerous others provide alternative therapeutic approaches [317,318,319,320].

In recent years, substantial advancements have been made in the fields of sensoring, imaging, and drug delivery systems utilizing MOFs [321]. These MOFs have demonstrated notable progress, offering advantages such as high porosity and extensive surface area, which enable efficient loading of drugs [321]. Additionally, they possess the ability for convenient functionalization and exhibit favorable qualities of biocompatibility and biodegradability, enhancing the effectiveness of drugs within living organisms and improving their availability [322]. The significant surface area and adjustable nanoporous structure attribute to the utilization of MOFs as biomarkers [323]. 

MOF-based commercial sensors have recently gained significant interest. Homayoonnia et al. developed a sensor device based on [Cu_3_(TMA)_2_(H_2_O)_3_]n in Cu-BTC nanoparticles (TMA: trimethylamine; BTC: triphosgene (bis(trichloromethyl) carbonate)) for volatile organic compound (VOC) detection [324]. Particularly, they examined their sensor in several analytes, such as ethanol, isopropanol, acetone, methanol, etc., with very promising reaction times and great linearity [325]. Several other studies have focused on MOF-based sensors for early detection of lung cancer biomarkers [326]. 

Liu et al. introduced a biocompatible nanoscale zirconium–porphyrin metal–organic framework (NPMOF)-based IGTS (ion-gated transistors), which is synthesized using a microemulsion technique and carefully controlled conditions [327]. This NPMOF demonstrates a remarkable porphyrin content of 59.8%, enabling remarkable fluorescent imaging and photodynamic therapy (PDT). The unique 1D channel structure within the MOF allows for a high doxorubicin loading capacity of 109% and pH-responsive controlled release for chemotherapy. Through laser irradiation, the fluorescence-guided chemotherapy and PDT dual system successfully directs the concentration of NPMOFs towards cancerous sites, facilitating doxorubicin release, while exhibiting minimal toxicity in normal tissues, thus acting as a theranostic apparatus [327]. 

Moreover, Wang et al. presented a method for synthesizing nanoscale MOF nanoparticles 50 nm, incorporating gadolinium (Gd^3+^) and europium (Eu^3+^) as metallic nodes. These nanoparticles are easily coated with a silica layer [328]. The resulting Eu, Gd-NMOF@SiO_2_ nanoparticles exhibit low toxicity, strong fluorescence, and demonstrate high longitudinal and transversal relaxivities on a 7T magnet. To target tumors specifically, the nanoparticles were conjugated with a peptide sequence known for its high affinity towards integrin ανβ3. Intratumoral or intravenous administration of Eu, Gd-NMOF@SiO_2_ nanoparticles leads to simultaneous signal enhancement and signal attenuation on T1- and T2-weighted images, respectively. These findings indicate the promising potential of NMOFs as a novel contrast agent capable of providing dual mode T1-T2 contrast imaging in MRI [328].

In the study of Ranjbar et al., a new and efficient approach was presented for producing Zn MOF nanocomposites containing encapsulated β-estradiol [329]. The Zn MOF nanocomposites were synthesized using Zn(OAc)_2_·2H_2_O and 2,6-pyridine dicarboxylic acid ammonium as the organic ligand. They focused on encapsulating β-estradiol, a crucial type of estrogenic compound widely utilized in the treatment of prostate cancer and breast cancer. The utilization of Zn MOF nanocomposites, characterized by their remarkable porosity and substantial total pore volume has offered an effective framework for accommodating β-estradiol and similar poorly soluble drugs. The maximum release of the drug reached approximately 82% after 8 h at pH = 8.9 [329]. 

In 2023, Meng et al. presented the development of heterostructures consisting of Ti-based MOFs that serve as a platform for in situ assembly of silver nanoparticles (AgNPs) [330]. Under ultrasound irradiation, the AgNPs effectively capture activated electrons from the MOF heterostructure, leading to a reduction in surrounding O_2_ and the generation of superoxide radicals. Simultaneously, the activated holes in the heterostructures promote the oxidation of water (H_2_O) to produce hydroxyl radicals. This synergistic effect enhances the therapeutic efficiency of sonodynamic therapy (SDT). By employing this structure as a mediator for SDT, they also demonstrated successful eradication of A549 lung cancer cells located beneath a 2 cm tissue barrier [330]. 

Furthermore, Zhao et al. developed a lanthanide-doped conversion nanoparticle and Mn MOF (DUCNPs-MnMOF) nanocarrier that demonstrated efficient loading and delivery of a cytotoxic antitumor agent (FOE) [331]. By combining the pH-responsive and peroxidase-like properties of Mn MOFs with the unique optical features of DUCNPs, the DUCNP@Mn-MOF/FOE exhibited synergistic chemodynamic and chemotherapeutic effects. Moreover, this nanosystem demonstrated responsiveness to the tumor microenvironment and displayed excellent tumor-targeting capabilities. As a result, the nanosystem demonstrated remarkable selectivity and efficient delivery of drugs, making it a promising candidate for cancer treatment. In a breast cancer mouse model, the nanosystem effectively suppressed tumor growth while maintaining low levels of toxicity. Therefore, this innovative nanosystem holds great potential as a theranostic platform for combining multiple diagnostic and therapeutic approaches in the treatment of tumors [331]. In their thorough review, Moharramnejad et al. conducted an extensive examination, highlighting the latest advancements of MOFs in the field of cancer treatment. The review encompasses their applications in photodynamic and photothermal therapy, along with their utility as imaging platforms [332]. Also, Chen et al. focused on inducing targeted VEGF-triggered release of an anticancer drug from aptamer-functionalized metal–organic framework nanoparticles and, particularly, amino-triphenyl dicarboxylate-bridged Zr^4+^ metal–organic framework nanoparticles [333]. Table 13 summarizes the main information obtained from the aforementioned studies. 

### 6.2. MOFs in Diabetes and Wound Healing

Diabetes is a long-term disease that presents a substantial global health concern. It is a metabolic disorder resulting from insufficient production or utilization of insulin. Insulin plays a crucial role in maintaining the equilibrium of blood sugar levels by aiding the absorption of glucose from fats and proteins. Hence, there is a need to develop a highly effective and responsive technique to detect insulin in serum [334]. 

In 2021, Adeel et al. synthesized Co-based two-dimensional (2D) metal nanosheets and developed a disposable glucose sensor possessing excellent redox activity in both neutral and alkaline environments [335]. This sensor demonstrated the ability to catalytically oxidize glucose, allowing for the non-enzyme detection of glucose in diluted human-plasma samples. Furthermore, copper-based MOF nanosheets displayed horseradish peroxidase-like activity, enabling direct detection of H_2_O_2_ and glucose fluorescence sensing with glucose sensing (Gox) [335].

Monitoring blood glucose levels daily has emerged as a significant global concern due to its direct impact on human health. So, in their study, Wang et al. conducted the synthesis on Ni/Co alloy nanoparticles encapsulated within graphitized carbon by subjecting a bimetallic (Ni and Co) metal–organic framework to pyrolysis at 800 °C in a nitrogen atmosphere [336]. Human blood samples were employed to assess the serum glucose levels utilizing electrodes modifies with Ni,Co-C composite. The sensor exhibited a linear detection range of 0.05 μM to 4.38 mM, with an optimized voltage of 0.50 V resulting in a detection limit of 0.2 μM. Furthermore, the sensor demonstrated excellent repeatability and long-term stability [336]. 

Zhang C. et al. constructed a glucose-responsive delivery system enclosing insulin (Ins) and glucose oxidase (Gox) within ZIF-8 nanocrystals as a means of encapsulation [337]. Glucose oxidase enzymatically converted glucose into gluconic acid, leading to a decrease in the local pH. This acidic environment triggered the breakdown of MOFs and enhanced the solubility of insulin, resulting in an accelerated release of the hormone. In vivo experiments demonstrated that a single injection of this formulation-maintained blood glucose levels in mice with type 1 diabetes for an extended period without the risk of hypoglycemia [337]. 

In 2022, Li et al. indicated that MOF Zn(BTC)_4_ can effectively encapsulate epigallocatechin gallate (EGCG) [338]. The sustained release of EGCG from EGCG-MOF-Zn(BTC)_4_ resulted in decreased expression of inflammatory factors and significant expression of inflammatory response in macrophages induced by lipopolysaccharide. In diabetic mice, wound healing experiments demonstrated that the addition of EGCG to MOF Zn(BTC)_4_ slowed down the degradation of EGCG, enabling a gradual release and prolonging its therapeutic effects. Treatment with this structure significantly reduced inflammation in the skin wound tissue of diabetic mice [338]. 

In another study, Zhang P. et al. conducted the modification of a copper-based MOF, called HKUST-1, for diabetic wound healing [339]. The inclusion of folic acid contributed to the stabilization of the MOF by enhancing its hydrophobicity and reducing the surface area. Consequently, this led to the controlled release of copper ions, mitigating their cytotoxic effects and facilitating diabetic wound healing [339]. Table 14 provides a comprehensive overview of the key findings extracted from the studies mentioned above.

### 6.3. MOFs in Brain and Neurological Disorders

Altered neuroanatomy, as well as abnormal secretion, uptake, and metabolism of specific biomarkers, can contribute to various neurological disorders. Detecting abnormal changes is essential for understanding treatment effectiveness and disease progression [340]. Researchers are extensively studying imaging agents based on MOFs for diagnosing brain cancer. These agents utilize the enhanced permeability and retention effect for passive targeting, or they bind to tumor-specific receptors for active targeting, allowing them to accumulate preferentially in tumor tissues. The presence of paramagnetic metal ions, such as Fe^3+^ and Mn^2+^, at the center of the MOFs’ structure makes them promising candidates as contrast agents for magnetic resonance imaging [341,342]. Pan et al. developed a bimetallic zeolitic imidazolate framework called mn-ZIF-8 to detect glioma, a type of brain tumor. This framework exhibited excellent biocompatibility, mainly attributed to the introduction of Mn^2+^ ions, which had significantly lower toxicity compared to Gd^3+^- based contrast agents commonly used in diagnostics [342]. 

The levels of dopamine, a critical neurotransmitter in the body, have a strong correlation with both the onset and progression of Parkinson’s disease. Ko et al. systematically investigated the electrochemical behavior of a glassy carbon electrode after being modified with two-dimensional layered conductive MOFs for the detection of dopamine, ascorbic acid, uric acid, and serotonin [343]. The enhancement in electron transfer was attributed to electrostatic forces. The electrodes that were modified with MOFs exhibited exceptional sensitivity towards analytes and provided good signal resolution [343]. 

There is a pressing need for the early and precise identification of Aβ oligomers to effectively evaluate patients with Alzheimer’s disease. Miao et al. proposed a novel dual-signal sandwich electrochemical immunosensor for the detection of Aβ [344]. The immunosensor employed Uio-66 modified with polyaniline for improved conductivity, and methylene blue was used as the signal label for anti-Aβ. Under optimized conditions, this immunosensor demonstrated satisfactory analytical performance. The electrochemical signals mediated by Cu-Al_2_O_3_-g-C_3_N_4_-Pd and Uio-66@PANI were utilized for Aβ detection. By employing these two parallel measurements, the biosensor not only enhanced the accuracy and efficiency of detection, but also minimized errors [344]. Table 15 offers a detailed summary of the primary data gathered from the previously referenced studies.

### 6.4. Toxicity Issues of MOFs

Considerable attention has been focused on investigating the potential harmful effects of MOFs in biological systems. Consequently, concerns have arisen regarding the compatibility of MOFs with biological system, necessitating a comprehensive understanding of the potential risks associated with their applications [345]. In vitro toxicity studies have proven highly advantageous due to their cost-effectiveness, rapidity, absence of animal involvement, and ability to facilitate high throughput screenings. Consequently, MTT colorimetric assay and cell counting have been employed to evaluate the toxicity of MOF nanoparticles. 

The metal ions employed during the fabrication of MOFs are typically of nanoscale dimensions and possess limited biodegradability. MOFs incorporating toxic metals such as arsenium and cadmium and can potentially lead to significant health issues due to the inherent toxicity of these metal constituents [346]. Consequently, when designing MOFs intended for drug delivery or other therapeutic purposes, it is advisable to utilize metals that are essential nutrients for the body, such as zinc and iron. The organic linkers employed in the synthesis of MOFs primarily consists of carboxylates, phenolates, amines and phosphonates, which are widely utilized. It is important to consider the potential health risks associated with these linkers, as MOFs are expected to degrade into their constituent materials over time. The characteristics of these linkers may give rise to significant health concerns, emphasizing the need for thorough evaluation and understanding of their potential effects [347]. 

### 6.5. Limitations in Biomedical Applications

While substantial advancements have been accomplished in laboratory investigations, there are still significant hurdles to overcome in the biomedical utilization of MOF-based composites. Foremost among these challenges is addressing the toxicity of MOFs prior to their clinical implementation. The toxicity of MOFs is not solely influenced by their composition, morphology, size, and robustness, but also by the resilience of living tissues, owing to the wide-ranging structures and types of metal–organic frameworks and the complex inner biological milieu. Thus, a comprehensive evaluation of the toxicity of various MOFs is imperative. Current research on MOF toxicity has primarily focused on immediate in vivo or acute toxicity studies, neglecting the crucial aspect of long-term toxicity, which is vital for considering MOF scaffolds as potential candidates for biomedical applications [348]. Moreover, employing endogenous and/or bioactive molecules as ligands and utilizing metal ions possessing increased biocompatibility, like iron, calcium and zinc, as metal nodes for developing effective metal–organic frameworks can be advantageous in avoiding MOF toxicity [349,350,351]. 

Moreover, hindering MOF agglomeration and early elimination during circulation poses a supplementary impediment. Excessive accumulation could lead to further noxious side impacts, while early disposal hampers optimal MOF efficacy [352]. Surface functionalization and size regulation have been employed to confront these issues. 

Lastly, there is a need for comprehensive studies on the degradation mechanisms, as well as pathways, of MOFs in vivo. Monitoring the persistent absorption, distribution, metabolism, and excretion pathway is crucial to gaining theoretical support and understanding MOF accretion in several tissues resulting from numerous administrations. Notably, the use of MOF nanocarriers for gene protection and delivery has garnered considerable attention. The efficiency of siRNA-mediated gene inhibition techniques hold promise for multiple untreatable disorders like hereditary diseases, cancer, Alzheimer’s and Parkinson’s disease, and diabetes [353,354]. Furthermore, the application of MOFs in immunotherapy represents a promising avenue for ailment therapies [355].

## 7. Future Perspectives

It is important for MOFs to be improved regarding their degradation efficiency. Deeper study is necessary to better understand the involved adsorption mechanisms, which is crucial for the design of multifunctional metal–organic frameworks characterized by enhanced elimination capability [289]. With regard to the high cost of organic ligands used in MOF synthesis, it is also very important for costs to be decreased through several strategies, such as the recycling of organic ligands. Furthermore, the form and consistency of MOFs should be improved in the near future. 

Through several studies, it is widely supported that it is crucial to conduct comprehensive research to assess, as well as endorse the effectiveness of water-stable metal–organic frameworks and their derivatives. Advanced studies should focus on the creation of water-stable MOFs for water treatment applications, aiming to resolve the technical hurdles stemming from the lack of stability of MOFs in aquatic environments, which limits their practical utility. 

The reliability and robustness of MOFs within more complex settings, like acid/base solutions and the interactions among different linkers and metal ions to fabricate acid- and base-resilient metal–organic frameworks also need optimization, and ecofriendly procedures are considered mandatory. 

In biomedical applications, the toxicity of MOFs is still under investigation, particularly when these applications might be exploited in clinical routine. Thus, in order to thoroughly assess MOF toxicity, comprehensive in vivo investigations and prolonged monitoring of tissue accumulation are critically essential.

The problem of MOF agglomeration might be solved through surface functionalization and size regulation. Regulating synthesis routes or employing physiochemical approaches enables effective control of MOF diameter within the nanoscale range, while surface functionalization using polymers, supramolecular macrocycles, and additional substances allows for the modulation of surficial charge and binding interactions. 

Moreover, despite of the fact that several studies have employed integrated imaging methods for monitoring MOF degradation, a more in-depth analysis of their degradation mechanisms is required.

To summarize, despite the fact that there are enduring difficulties in MOFs’ biomedical applications, the tremendous advancements achieved thus far serve as a fundamental reference towards studying MOF toxicity, biodegradability, and degradation mechanisms. With their multidisciplinary advantages, we envision further advancements in the clinical use of MOFs to improve human health in the near future.

## 8. Conclusions

Based on the comprehensive literature review, it can be concluded that MOFs possess unique characteristics that make them highly suitable for removing pollutants from water. In recent years, there has been burgeoning concern/curiosity regarding researching MOF applications due to their increased specific surface area, porosity, and robustness under various conditions, while they sustain their structural durability even after removing guest molecules. The hydrothermal/solvothermal approach is frequently employed for MOF synthesis, involving transition metal precursors like copper, magnesium, iron, zinc, and dicarboxylic acid compounds as organic ligands. Recent advancements in MOF synthesis have enabled the introduction of functional groups within their structure and the development of magnetic metal–organic frameworks. Such approaches improve upon traditional synthesis routes and offer advantages over conventional adsorbents. Notably, biomass-derived MOFs and mechanochemical synthesis allow for the utilization of cost-effective and renewable precursors, promoting green and biocompatible MOF production. These novel synthesis methods result in enhanced MOF structures and properties, leading to reduced reaction times and improved yields. 

Additionally, MOFs remove pollutants through various adsorption mechanisms, including electrostatic and acid–base interactions, H-bonding, π–π stacking, and hydrophobic interactions. The effectiveness of MOFs as solid adsorbents is contingent upon factors like contact time, adsorbent dosage, pollutant concentration, pH, and ionic strength. The procedure’s economic feasibility could be enhanced through utilizing organic materials as precursors and minimizing the use of chemical solvents. However, certain limitations hinder the widespread application of MOFs. The enhanced organic ligands’ cost adversely affects the economic efficiency of metal–organic frameworks, while their low water stability limits their operational range. Concerns regarding the leaching of metal ions into solution pose potential risks of secondary water pollution. While MOFs have shown success in simulated wastewater treatment, improvements are needed in terms of selectivity, particularly for continuous membrane applications. Also, scientific research attempts should focus on testing MOF performance in real wastewater on a larger scale to evaluate factors like H_2_O flow, mass transfer, and pollutant load. Further studies are also required to assess environmental toxicity and lifecycle consequences of metal–organic frameworks, particularly their elimination after usage. In spite of these difficulties, metal–organic frameworks hold great potential as a low-cost and reliable option for wastewater remediation.

A detailed review and analysis of the recent advancements in MOF-based composites for biomedical applications was also conducted. These applications include cancer treatment, diabetes therapy, and wound healing, as well as brain and neurological disorder therapy. The continuous fabrication of advanced MOF-based nanoplatforms has been driven by the increasing demands in this field. Additionally, researchers have focused on creating MOF-based materials that exhibit precise targeting capabilities and minimal toxic side effects in biomedical applications. Notably, the multifunctional nature of metal–organic frameworks in biomedicine stems from their versatile structure and morphology, impressive specific surface areas, increased porosity and crystallinity, exceptional loading efficiency, high thermal and chemical stability, and adjustable bonds. Despite noteworthy advancements in laboratory research, there are still significant challenges that require attention for the practical utilization of MOF-based composites in biomedical applications.

## Figures and Tables

**Table 4:** MOF features fabricated using the mechanochemical approach.

Type of MOF	Precursor Materials		Solvent Type	Experimental Parameters	Comments	Ref.
	Organic Ligand	Metal Salt				
ZIF-62	C_3_H_4_N_2_	ZnO	SF	G, 30 min	Mixed metal MOFs	[95]
ZIF-8	C_4_H_6_N_2_	ZnO	SF	BL, 720 min	Well-dispersed MOFs	[96]
ZIF-8	C_4_H_6_N_2_	Zn(OH)_2_	SF	G, 60 min	Rapid fabrication of MOFs	[97]
ZIF-8	C_4_H_6_N_2_	ZnCO_3_	SF	BL, 720 min	MOFs with increased specific surface area	[98]
ZIF-8/ ZIF-67	C_4_H_6_N_2_	Zn(CH_3_COO)_2_ 2H_2_O/Co(CH_3_COO)_2_ 2H_2_O	SF	BL, 120 min	Water-robust MOFs	[99]
MOF-74	C_8_H_6_O_6_	Mg(NO_3_)_2_ 6H_2_O	SF	G, 5 min	MOFs with increased crystallinity and specific surface area	[100]
MOF-74	C_8_H_6_O_6_	ZnO	DMF	G, 70 min	MOFs with high crystallinity and porosity	[101]
Ni-UiO-66	C_8_H_6_O_4_	Ni(NO_3_)_2_ 6H_2_O	SF	BL, 10 min	MOFs with enhanced catalytic efficiency in H_2_ generation reactions	[102]

Note: SF = solvent-free; BL = ball-milling; G = grinding.

**Table 5:** Comparative assessment of the mechanochemical and traditional solvothermal approaches.

Solvothermal Approach	Mechanochemical Approach
Multistage procedure.	One-stage procedure.
Elevated thermal energy demand.	Room temperature.
Massive volumes of liquid waste produced.	Negligible production of liquid wastes.
Large amounts of potentially noxious solvents consumed.	Solvent-free or limited amounts of solvents used.
Increased costs (multiple steps, solvents, post-waste treatment).	Low cost.
Basic equipment requirements.	Distinctive mills and grinders requirements.
Products with enhanced crystallinity.	Potential development of amorphous phases.
Ease in precise control of the reaction procedure.	Difficulties in precise control of the reaction procedure.
Development of pure products.	Potential impurities during milling.

**Table 6:** MOF features synthesized using the ambient temperature stirring approach.

Type of MOF	Precursor Materials		Solvent Type	Comments	Ref.
	Organic Ligand	Metal Salt			
ZIF-8	C_4_H_6_N_2_	Zn(NO_3_)_2_	CH_3_OH	MOFs with adjustable particle size	[106]
ZIF-8	C_4_H_6_N_2_	Zn(NO_3_)_2_ 6H_2_O	Distilled water	Two-dimensional bimetallic MOFs with flake-like nanosheet shapes	[107]
MOF-801	Tetrakis (4-carboxyphenyl) porphyrin	ZrCl_4_ 8H_2_O	N/A	MOFs with adjustable particle size	[108]
BZIF-8-B	C_4_H_6_N_2_	Zn(CH_3_COO)_2_ 2H_2_O	Distilled water	Multiphase biomolecular MOFs	[104]
PCN-224-RT	Tetrakis (4-carboxyphenyl) porphyrin	Zr(OBu)_4_	DMF	Robust and functional porphyrinic MOFs for the encapsulation of metallic nanoparticles	[109]
ZIF-67-NS	C_4_H_6_N_2_	Co(NO_3_)_2_ 6H_2_O	DMF	Ultrathin 2D MOFs with exceptional adsorption efficiency towards As^3+^	[103]
Fe-Co MOF	C_8_H_6_O_4_	Co(NO_3_)_2_ 6H_2_O/FeCl_3_ 6H_2_O	DMF	Nanosheet-shaped MOFs possessing electrocatalytic attributes	[110]
NKMOF-8-Br	C_5_H_2_N_4_	CuI	C_2_H_3_N	Isostructural MOFs with 3D porous (ultra μm) network	[111]

**Table 7:** MOF features synthesized using the electrospinning approach.

Type of MOF Product	Precursor Materials	Utilized Conditions during Synthesis	Comments	Ref.
MOF nanofibers	PVP, ZIF-8	5 kV, 0.35 mL/h	Hierarchical MOF nanofibers with increased porosity	[115]
MOF nanofibers	PAN, ZIF-8	18 kV, 1 mL/h	Robust MOF fibers with exceptional reusability regarding the elimination of various pollutants	[116]
MOF nanofibers	PAN, ZIF-8	20 kV, 0.5 mL/h	MOF fibers with enhanced adsorption efficiency in the elimination of ionic dyes	[117]
MOF nanofibers	PVA, Ni MOF	20 kV, 0.1 mL/h	MOF fibers used for CH_4_ adsorption	[118]
MOF carbon nanofiber	PAN, ZIF-8	25 kV, 0.48 mL/h	Hierarchical MOF nanofibers with increased porosity	[119]
MOF membranes	PVP, ZIF-8	12 kV	ZIF-8 membranes lacking defects	[120]

Note: PVP = polyvinylpyrrolidone; PAN = peroxyacetyl nitrate; PVA = polyvinyl acetate.

**Table 8:** MOF-based carbon materials obtained using the carbonization approach.

Type of MOF Product	Precursor Materials	Utilized Conditions during Synthesis	BET Surface Area (m^2^/g)	Ref.
Porous carbon	Zn(bdc)(ted)_0_._5_	310 °C 60 min N_2_	1270	[126]
Porous carbon	MOF-5	1000 °C 120 min Ar	1884	[127]
Bimetallic porous N-doped carbon	ZIF-67	675 °C 180 min Ar	244	[128]
Bimetallic porous carbon	ZIF-8	800 °C 360 min N_2_	1439.5	[129]
Hierarchical porous carbon	Zn_3_(fumarate)_3_(DMF)_2_	1100 °C 480 min Ar	1834	[130]
N-doped carbon nanotubes	ZIF-8	350–900 °C	1323.5	[131]
Carbon composite membrane	MIL-125-NH2	800 °C 120 min N_2_	266	[132]
N-doped porous carbon	ZIF-8	900 °C 120 min N_2_	3077	[133]

**Table 9:** Comparison of the existing synthetic MOF approaches [51,78,112,122].

MOFs’ Synthetic Approach	Benefits	Drawbacks
Solvothermal/ hydrothermal	Production of simple crystals. Basic equipment needed. Production of materials characterized by enhanced crystallinity and purity. Relatively easily controllable reaction conditions.	Multistage procedure. High energy consumption due to increased temperatures requirements. Large quantities of solvents required. Post-waste requirements (not cost efficient).
Microwave	Short reaction times. Increased product yields. Tunable MOF attributes. Production of materials with enhanced crystallinity and purity. Low energy consumption. Limited post-waste treatment requirements and byproducts.	Sometimes difficult to control the reaction conditions. Nonreproducible reaction conditions.
Sonochemical	Fast reaction times. Ecofriendly. Limited energy consumptions. Production of materials with exceptional crystallinity.	Limitations in the control of the utilized temperatures. Poor control ability of MOFs’ pore structure.
Mechanochemical	One-stage procedure. Room temperature conditions. Limited production of postreaction byproducts. Possibility for synthesis in the absence of solvents. Low-cost approach.	Special equipment requirements (mills and grinders). Development of amorphous phases impacting the produced material’s degree of crystallinity. Possibility for impurities to be observed. Sometimes difficult to control the reaction conditions.
Ambient temperature stirring	Rapid crystallization. Chemically and thermally robust materials. Low temperature requirements. Simple molecular crystals can be obtained.	Increased temperatures may be required during the utilization of kinetically inert metal ions. Products characterized by limited purity due to incomplete solvents’ elimination.
Electrospinning	Facile and effective. Adjustable. Development of materials possessing increased specific surface areas.	Not easily scaled up. Risk of degradation when polymers are utilized.
Carbonization	Possibility to adjust the features of the obtained materials by controlling carbonization conditions.	Increased energy consumption.
Electrochemical	Ability to produce materials continuously. Production of materials with increased solids content. Ease in scale-up applications. Rapid reaction procedures. Moderate reaction conditions.	Continuous electrical contact between the power source and electrode is required in order to guarantee continuous material production. Limitations regarding the solubility of inert organic linkers. Poorly studied cathodic reaction mechanism.

**Table 10:** Selected materials for heavy metal elimination.

Type of MOF	Synthetic Approach	Heavy Metals Tested	Adsorption Efficiency (mg/g)	Kinetic Model	Isotherm Model	Reusability	Ref.
Fe_3_O_4_-Zr MOF	Coprecipitation	Hg^2+^, Cd^2+^ Pb^2+^	431 393 397	Pseudo-second-order	Langmuir	3 cycles	[189]
UiO-66-Cl UiO-66-S	Solvothermal	Fe^3+^	480	Pseudo-second-order	-	6 cycles	[182]
Zr MOF	Solvothermal	Cu^2+^	125	-	-	-	[178]
UiO-66-EDA	Michael addition reaction	Pb^2+^ Cd^2+^ Cu^2+^	243.9 217.4 208.3	Pseudo-second-order	Langmuir	4 cycles	[175]
ZIF-67	Facile method	Cu^2+^ Cr^6+^	200.6 152.1	-	-	5 cycles	[40]
ZIF-67@Fe_3_O_4_@ESM composite	Ultrasound-assisted method	Cu^2+^	344.8	Pseudo-second-order	Langmuir	5 cycles	[202]
PCN-221	Solvothermal	Hg^2+^	233	Pseudo-second order	Langmuir	3 cycles	[196]
[Zn_2_(oba)_2_(bpfb)] (DMF)_5_ (TMU-23)	Solvothermal	Pb^2+^	434.7	Pseudo-second-order	Langmuir	3 cycles	[176]
{[(Zn (ADB)L_0_._5_] 1.5DMF}_n_	Solvothermal	Pb^2+^	463.5	Pseudo-second-order	Langmuir	3 cycles	[203]
Melamine-modified MOFs	Thermal-promoted method	Pb^2+^	122	Pseudo-second-order	-	5 cycles	[184]
Other adsorbents							
*Eragrostis tef* activated carbon	Pyrolysis	Pb^2+^	43	Pseudo-second-order	-	-	[204]
Hydrochar	Solvothermal	Pb^2+^	38.3	Yoon–Nelson	-	-	[205]
Multi-wall CNTs	-	Cu^2+^ Zn^2+^ Fe^2+^ Pb^2+^	142.8 250 111.1 200	Pseudo-second order	Langmuir	-	[206]
GO	Hummer	Pb^2+^	55.8	Pseudo-second-order	Langmuir	-	[207]
Fly ash	-	Cd^2+^	124.9	Pseudo-second-order	Langmuir	-	[208]
Bottom ash	-	Cd^2+^	23.3	Pseudo-second-order	Langmuir	-	[209]
HAp	Ultrasonic	Cu^2+^ Zn^2+^ Cd^2+^	272 285 304	Pseudo-second-order	Freundlich	-	[209]
HAp-HA	-	Cu^2+^	35.2	Elovich	Sips	4 cycles	[210]

**Table 11:** Selected materials for organic dye elimination.

Type of MOF	Synthetic Approach	Organic Dye Tested	Adsorption Efficiency (mg/g)	Kinetic Model	Isotherm Model	Reusability	Ref.
MOF 8	Sol–gel	Malachite green	613	Pseudo-second-order	Langmuir	3 cycles	[226]
Ca-Al MOF	Ion exchange	Malachite green	84.5% (elimination capacity)	Modified pseudo-first-order	-	-	[233]
Fe MOF	Hydrothermal	Alizarin red	176.7	Pseudo-first-order	Langmuir	-	[234]
Fe MOF	Hydrothermal	Rhodamine B	90% (elimination capacity)	Pseudo-first-order	-	4 cycles	[238]
Bi MOF	-	Rhodamine B	98% (elimination capacity)	Pseudo-first-order	-	4 cycles	[239]
Ni(II)-doped MIL-101(Cr)	Hydrothermal	Congo red/ methyl orange	1607.4 651.2	-	-	4 cycles	[245]
{[Zn(1,3-BDC)L]•H_2_O}_n_	Hydrothermal	Amido black 10B, methyl orange, direct red 80	2402.82 (AB), 744 (MO), 1496.34 (DR)	Pseudo-second-order	Langmuir/Sips	5 cycles	[91]
ZIF-67@ Fe_3_O_4_@ESM	Sonochemical	Basic red 18	250.8	Pseudo-second-order	Langmuir	5 cycles	[202]
Ni-MOF-199	Solvothermal	Methylene blue	765	Pseudo-second-order	Langmuir	-	[237]
Ni-MOF-199	Solvothermal	Methylene blue	798	Pseudo-second-order	Langmuir	-	[237]
ZIF-67	-	Active red X-3B	100% (elimination capacity)	-	-	-	[40]
ZIF-67@wood composite	Carbonization	Congo red Methylene blue	1117.03 (CR) 805.08 (MB)	Pseudo-second-order	Langmuir	20 cycles	[242]
Other adsorbents							
Rice husk activated carbon	Carbonization	Rhodamine B	478.5	Pseudo-second-order	Langmuir	-	[246]
Orange peel activated carbon	Microwave pyrolysis	Malachite green	28.5	-	-	-	[247]
Activated carbon aerogel	-	Methylene blue	416.7	Pseudo-second-order	Langmuir	3 cycles	[248]
GO-HAp	Sonochemical	Congo red/Trypan blue	48.5 41.0	Pseudo-second-order	Langmuir	4 cycles	[249]
Magnetic xanthate modified chitosan	-	Methylene blue/Safranin O	197.8 169.8	Pseudo-second-order	Sips	-	[250]
GO-activated carbon		Methylene blue/Crystal violet	147.0 70.0	Pseudo-second-order	Freundlich Langmuir	5 cycles	[251]

**Table 12:** Selected materials for antibiotics’ elimination.

Type of MOF	Synthetic Approach	Antibiotics’ Tested	Adsorption Efficiency (mg/g)	Kinetic Model	Isotherm Model	Ref.
PCN-222	Solvothermal	Chloramphenicol	370	Pseudo-second-order	Langmuir	[259]
MOF–chitosan composite	Solvothermal	Tetracycline	495	Pseudo-second-order	Langmuir	[266]
Alg@MOF-rGO	-	Tetracycline	43.8	Pseudo-second-order	Langmuir	[264]
Alg@MOF-rGO	-	Ciprofloxacin	40.8	Pseudo-second-order	Langmuir	[264]
UiO-66		Tetracycline	145	Elovich	Sips	[262]
NU-1000	Solvothermal	Tetracycline	356	Elovich	Sips	[262]
MOF-525	Solvothermal	Tetracycline	807	Pseudo-second-order	Sips	[262]
α-Fe/Fe_3_C MOF composite	Solvothermal	Tetracycline	166.7	Pseudo-second-order	Langmuir	[258]
Fe MOF	Solvothermal	Tetracycline	714.3	-	-	[261]
Fe MOF	Solvothermal	Norfloxacin	346.6	-	-	[261]
CuCo/C-MOF-71	Carbonization	Ciprofloxacin	90% (elimination efficiency)	-	-	[263]
NH_2_-MIL-101-Fe		Metronidazole	90% (elimination efficiency)	Pseudo-second-order	Langmuir	[273]
Other adsorbents						
Biochar	Calcination	Tetracycline	297.90	Pseudo-second-order	Langmuir	[274]
Hydrochar	Hydrothermal carbonization	Sulfamethoxazole	740.6	Pseudo-second-order	Langmuir	[275]
Magnetic orange peel adsorbent	Microwave	Sulfamethoxazole	120	Pseudo-second-order	Redlich-Peterson	[276]
Hydrogel	Carbonization	Ciprofloxacin	106	Pseudo-second-order	Langmuir	[277]
Nanocellulose	Hydrolysis	Diclofenac	192	Pseudo-second-order	Halsey	[278]

**Table 13:** MOF-based devices for diagnosis and therapy of cancer disease.

Material	Function	Disease/Disorder	Ref.
Cu_3_(TMA)_2_(H_2_O)_3_]_n_ in Cu-BTC NPs MOFs	Sensor (VOC detection)	Lung cancer, etc.	[324]
Nanoscale zirconium–porphyrin metal–organic framework (NPMOF)-based IGTS (ion-gated transistors)	Fluorescent imaging/chemotherapy and photodynamic therapy (PDT)	Cancer	[327]
Eu, Gd-NMOF@SiO_2_ NPs	Imaging (MRI)	Cancer	[328]
Zn MOF	Drug delivery	Cancer	[329]
Ti-based MOF AgNPs	Sonodynamic therapy (SDT)	Cancer	[330]
Lanthanide-doped up conversion NPs and Mn MOFs (DUCNPs-MnMOF)	Drug delivery	Cancer	[331]
Amino-triphenyl dicarboxylate-bridged Zr^4+^ MOF nanoparticles	Drug delivery	Cancer	[333]

**Table 14:** MOF-based devices for wound healing and diagnosis of diabetes.

Material	Function	Disorder	Ref.
Co-based 2D metal nanosheets	Glucose sensor	Diabetes	[335]
Bimetallic (Ni and Co) MOF	Sensor	Diabetes	[336]
ZIF@Ins@GOx	Glucose responsive delivery system	Diabetes	[337]
Zn(BTC)_4_ MOF	Drug delivery–treatment	Wound healing	[338]
HKUST-1 (copper-based MOF)	Therapy	Wound healing	[339]

**Table 15:** MOF-based materials for neurological disorders.

Material	Function	Disorder	Ref.
Fe-MIL-88B-NH_2_-NOTA-DMK6240/MB	Drug delivery, MRI contrast material	Alzheimer’s disease	[341]
Μn-ZIF-8	Drug delivery, in vivo MRI	Glioma	[342]
2D MOFs	Electroanalytical device (voltammetric detection)	Parkinson’s disease	[343]
Cu-Al_2_O_3_-g-C_3_N_4_-Pd	Immunosensor	Alzheimer’s disease	[344]

## Data Availability

Not applicable.
